# Safe and efficient 2D molybdenum disulfide platform for cooperative imaging-guided photothermal-selective chemotherapy: A preclinical study

**DOI:** 10.1016/j.jare.2021.08.004

**Published:** 2021-08-11

**Authors:** Xin Li, Lingdan Kong, Wei Hu, Changchang Zhang, Andrij Pich, Xiangyang Shi, Xipeng Wang, Lingxi Xing

**Affiliations:** a Department of Gynecology and Obstetrics, XinHua Hospital Affiliated to Shanghai JiaoTong University School of Medicine, Shanghai 200092, China; b DWI-Leibniz-Institute for Interactive Materials e.V., 52056 Aachen, Germany; c Institute of Technical and Macromolecular Chemistry, RWTH Aachen University, 52074 Aachen, Germany; d College of Chemistry, Chemical Engineering and Biotechnology, Donghua University, Shanghai 201620, China; e Laboratory of Nanoscale Biosensing and Bioimaging, School of Ophthalmology and Optometry, School of Biomedical Engineering, Wenzhou Medical University, Wenzhou 325027, China; f CQM-Centro de Química da Madeira, Universidade da Madeira, Campus da Penteada, 9000-390 Funchal, Portugal; g Aachen Maastricht Institute for Biobased Materials, Maastricht University, 6167 RD Geleen, the Netherlands

**Keywords:** 2D molybdenum disulfide, Tocopheryl succinate, Multimode theranostics, Safe NIR irradiation, Selective chemotherapy

## Abstract

**Introduction:**

The striking imbalance between the ever-increasing amount of nanomedicines and low clinical translation of products has become the focus of intense debate. For clinical translation, the critical issue is to select the appropriate agents and combination regimen for targeted diseases, not to prepare increasingly complex nanoplatforms.

**Objectives:**

A safe and efficient platform, α-tocopheryl succinate (α-TOS) married 2D molybdenum disulfide, was devised by a facile method and applied for cooperative imaging-guided photothermal-selective chemotherapy of ovarian carcinoma.

**Methods:**

A novel platform of PEGylated α-TOS and folic acid (FA) conjugated 2D MoS_2_ nanoflakes was fabricated  for the cooperative multimode computed tomography (CT)/photoacoustic (PA)/thermal imaging-guided photothermal-selective chemotherapy of ovarian carcinoma.

**Results:**

The photothermal efficiency (65.3%) of the platform under safe near-infrared irradiation is much higher than that of other photothermal materials reported elsewhere. Moreover, the covalently linked α-TOS renders platform with selective chemotherapy for cancer cells. Remarkably, with these excellent properties, the platform can be used to completely eliminate the solid tumor by safe photothermal therapy, and then kill the residual cancer cells by selective chemotherapy to prevent tumor recurrence. More significantly, barely side effects occur in the whole treatment process. The excellent efficacy and safety benefits *in vivo* lead to the prominent survival rate of 100% over 91 days.

**Conclusion:**

The safe and efficient platform might be a candidate of clinical nanomedicines for multimode theranostics. This study demonstrates an innovative thought in precise nanomedicine regarding the design of next generation of cancer theranostic protocol for potential clinical practice.

## Highlights

• Safe and efficient platform of TOS married MoS_2_ is synthesized by judicious designed for multimode theranostics of ovarian carcinoma.
• A photothermal conversion efficiency of 65.3% of the platform is higher than that of other materials reported elsewhere.
• Highly efficient photothermal ablation under safe irradiation and significantly improved selective chemotherapy for tumor.
• Synergistic therapy, suppressed recurrence, and negligible side effects enable the prominent survival rate of 100% over 91 days for the tumor-bearing mice.
• A promising candidate for precise nanomedicines in clinical translation.

## Graphical abstract

**Figure:**
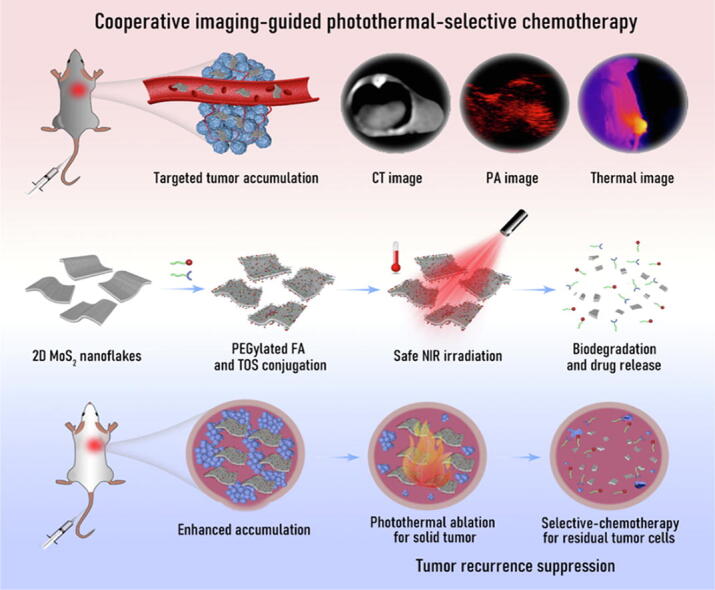

## Introduction

The accurate diagnosis and efficient treatment are necessary and critical for precise medicine [1], [2], [3]. Currently, for most tumors treatment, the imaging-guided surgical resection associated with postoperative chemotherapy is the best option in clinical practice. Ovarian carcinoma is one of the most lethal gynecological tumors, and 70% of patients are diagnosed at advanced stage because of lack of specific symptoms and exact imaging methods [4], [5]. Moreover, these patients will face radical operation, multiple chemotherapy courses and relapses. However, some patients with advanced cancer are no longer available for invasive surgery because of major risk or/and systemic chemotherapy due to severe side effects [6], [7], [8]. Therefore, it is extremely urgent to develop novel theranostic strategies with accurate images, highly efficient therapy and slight side effects, to meet the requirement of clinical operation.

In recent years, ever more complex theranostic platforms was reported continuously in preclinical research, but the success rate in clinical translation for these nanomedicine products remained low [9], [10], [11], [12], [13]. The striking imbalance between the ever-increasing nanomedicines and low clinical translation has become the focus of intense debate [14]. In the aspect of clinical translation, the critical issue is to select the appropriate therapeutic agents and combination scheme for targeted diseases, not to prepare increasingly complex nanomaterials [15], [16].

In 2020, Chen *et al.* [17] pointed out that compared to other strategies, the combination of photothermal therapy and currently approved chemotherapy is most likely to be the first to achieve clinical practice due to their distinct advantages. Likewise, the combined photothermal-chemotherapy has been explored to improve synergistic antitumor efficiency for solid tumors [18], [19], [20], [21], [22]. However, for most of photothermal agents, hindered by the insufficient tumor accumulation and low photothermal conversion efficiency (PCE), the operations were forced to increase the injection dosage or/and take excessively high near-infrared (NIR) irradiation (power of 1.0–3.0 W/cm^2^) to ensure tumor ablation effect [23], [24], [25], [26], [27]. According to the standard from the American National Standard Institute (ANSI) [28], these NIR irradiation are significantly higher than the safe maximal permissible exposure (MPE) of skin (e.g., 0.33 W/cm^2^ at 808 nm laser). Additionally, for drug-loaded carriers, the low loading efficiency, rapid drug leakage, nonspecific anticancer activity, and serious side effects limit their applications greatly [29], [30], [31], [32], [33], [34]. Accordingly, to promote the substantial progress of nanomedicine in clinical translation, it is necessary to design the safe and highly efficient platform for the combined photothermal-chemotherapy of tumors upon permitted laser irradiation.

Currently, 2D nanomaterials, as ideal photothermal agents, have attracted much attention in biomedical fields due to their high PCE, large ratio of surface area-to-volume, and easy surface modification [35], [36], [37]. In particular, 2D molybdenum disulfide (MoS_2_) with excellent biocompatibility and valuable biodegradability can be exploited as unprecedented nanotemplates to hybrid other functional elements for highly effective cancer theranostics [38], [39]. Moreover, a kind of vitamin E derivative, α-tocopheryl succinate (α-TOS), possesses excellent anticancer activity against various types of human cancers by inducing apoptosis, with no toxicity to normal cells [40], [41]. But the poor water solubility of α-TOS hindered their further applications [42]. According to these latest concepts of clinical translation, we firmly believed that the α-TOS married 2D MoS_2_ as a promising theranostic platform can be employed to achieve the compelling efficacy and safety benefits for cancer theranostics. Despite all this, to our knowledge, the similar system has not been designed and reported. Moreover, the comprehensive characterization of the performance and potential of theranostic platform in the biomedical application is also very critical.

**Fig. 1:**
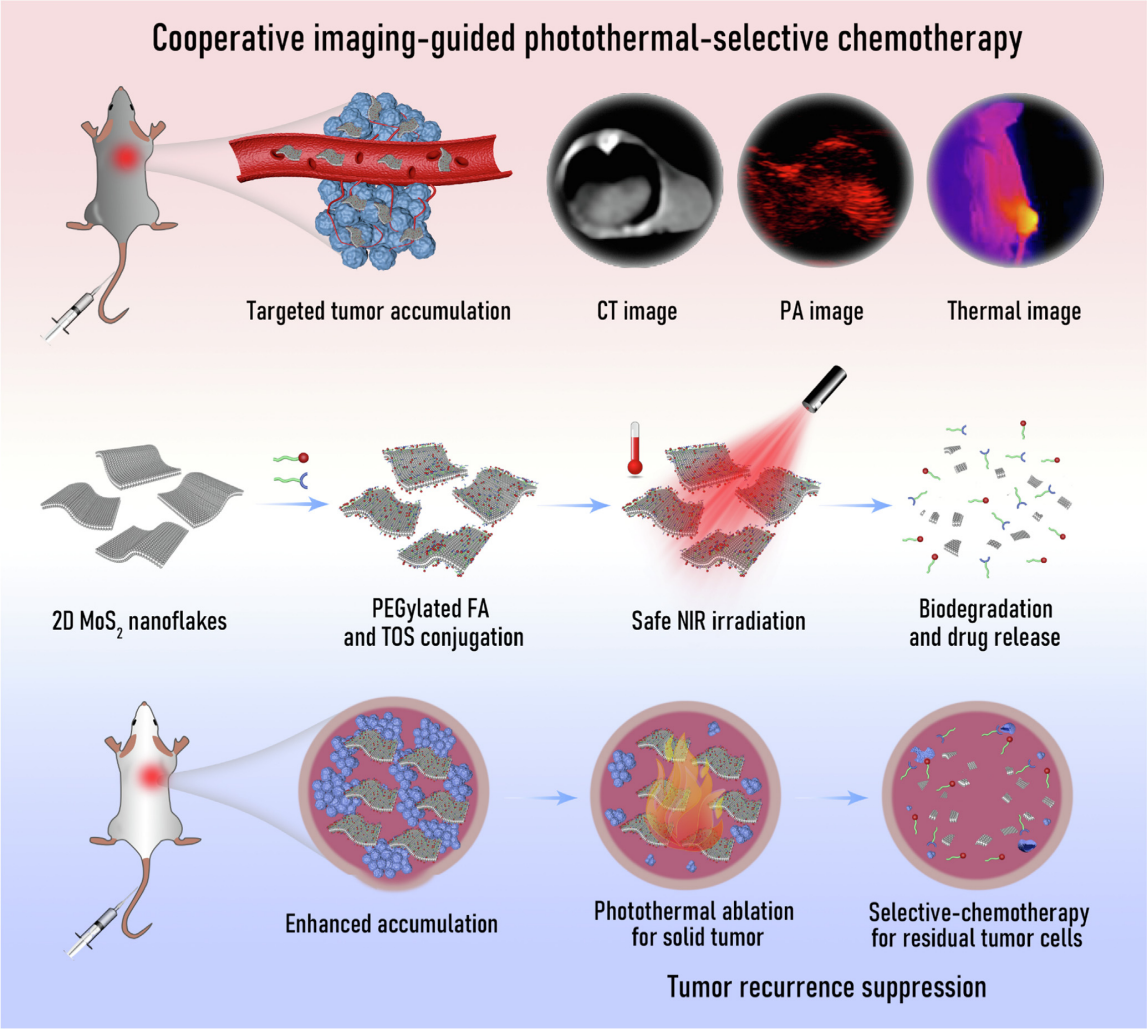
Schematic preparation of safe and highly efficient 2D MoS_2_-based platform for cooperative CT/PA/thermal imaging-guided photothermal-selective chemotherapy of ovarian carcinoma under permitted NIR irradiation: Efficient theranostics, recurrence suppression, prominent survival rate, and barely side effects.

Herein, for the first time, the safe and highly efficient platform, PEGylated α-TOS and folic acid (FA) conjugated 2D MoS_2_ nanoflakes (MoS_2_-PEG-TOS-FA, or MPTF for short), has been designed for cooperative multimode computed tomography (CT)/photoacoustic (PA)/thermal imaging-guided photothermal-selective chemotherapy of ovarian carcinoma (Fig. 1). The created MPTF exhibits a satisfactory PCE (65.3%) under safe NIR irradiation (808 nm, 0.3 W/cm^2^), which is much higher than that of other photothermal materials reported elsewhere (Table S1). Encouragingly, the covalently linked α-TOS renders MPTF selective and enhanced chemotherapy for ovarian cancer cells, while no significant toxicity to normal ovarian cells. Notably, a series of *in vivo* parameters, including targeted tumor tissue accumulation, multimode images, tumor elimination, recurrence suppression, survival ratio and time, systemic toxicity, blood biochemistry index, tissue distribution, biodegradation, and metabolism, were systematically characterized to evaluate the potential for clinical translation. By systemic injection, the MPTF could be employed to preoperatively locate ovarian tumor by multimode images, and then highly efficient photothermal therapy could be triggered to completely ablate the entire solid tumor under safe NIR irradiation, finally the locally infiltrating and metastatic cancer cells would be killed by selective chemotherapy to prevent recurrence. Based on the obtained results *in vitro* and *in vivo*, the developed nanoplatform using the appropriate agents (α-TOS married 2D MoS_2_) and the combination regiment (photothermal-selective chemotherapy) may be considered as the candidate in preclinical nanomedicine libraries for the safe and highly effective theranostics of ovarian carcinoma.

## Experimental

### Synthesis of MoS_2_ nanoflakes, LA-PEG-FA, LA-PEG-TOS and MPTF

2D MoS_2_ nanoflakes were synthesized by hydrothermal approach. Briefly, (NH_4_)_2_MoS_4_ powder (50 mg) dissolved in 10 mL water was stirred for 30 min, followed by ultrasound for 10 min to completely dissolve the powder. Subsequently, 0.227 mL of (N_2_H_4_)**^.^**H_2_O was added under stirring, and then furtherly sonicated for 30 min. The reaction mixture was transferred to a Teflon-lined stainless-steel autoclave with a volume of 50 mL. After that, the autoclave was placed in an oven at 200 °C for 10 h. Finally, the black solution was obtained after cooling down to room temperature, and then centrifugated (10,000 rpm, 10 min) and washed by water for 5 times to collect MoS_2_ nanoflakes.

Next, the lipoic acid (LA)-PEG-FA and LA-PEG-TOS were synthesized by 1-ethyl-3-(3-dimethylaminopropyl) carbodiimide hydrochloride (EDC) chemistry, respectively. Typically, FA (44.1 mg) or α-TOS (53.1 mg) dissolved in 3 mL DMSO was activated by EDC (96.0 mg, in 2 mL DMSO) under vigorously stirring for 30 min at room temperature, then NHS (57.5 mg, in 2 mL DMSO) was added and further stirred for 3 h. After that, BocNH-PEG-NH_2_ (200 mg, Mw = 2000) dissolved in 3 mL DMSO was added to the above solution and further stirred for 3 days, then 200 μL of HCl (3–4 M) was added and further stirred for 1–2 h to exfoliate the group of -Boc. The reaction mixture was then dialyzed against water (9 times, 2 L) using a dialysis membrane (MWCO = 1000) for 3 days, followed by lyophilization to obtain the product of NH_2_-PEG-FA or NH_2_-PEG-TOS. In addition, the LA (20.6 mg, in 3 mL DMSO) was activated by EDC (96.0 mg, in 2 mL DMSO) and NHS (57.5 mg, in 2 mL DMSO) respectively, according to the aforementioned protocol. After that, NH_2_-PEG-FA (244 mg) or NH_2_-PEG-TOS (253 mg) dissolved in 3 mL DMSO was added to the above solution and further stirred for 3 days. The reaction mixture was dialyzed using dialysis membrane (MWCO = 1000) and freeze-dried to achieve the final product of LA-PEG-FA or LA-PEG-TOS.

Finally, the MoS_2_ nanoflakes (50 mg) dispersed in 5 mL water were reacted with LA-PEG-FA (250 mg, in 3 mL water) and LA-PEG-TOS (250 mg, in 3 mL water) under stirring for 12 h. The reaction mixture was dialyzed using dialysis membrane (MWCO = 8000–14000) and freeze-dried to obtain the final product of MPTF. Also, MoS_2_-PEG-FA (MPF for short) and MoS_2_-PEG-TOS (MPT for short) were prepared as control samples under same experimental condition.

### In vitro cell culture and assays

SKoV3 cells (human ovarian cancer cell line) were regularly cultured and passaged for cytocompatibility, the images of cell cytoskeleton and nuclei, specific targeting, selective anticancer activity assays, combined photothermal-selective chemotherapy, anticancer mechanism analysis of different samples. See additional details in the supplementary material.

### Ethics statement

All animal experiments involving animals were conducted according to the ethical policies and procedures approved by the Ethical Committee of Shanghai XinHua Hospital (Approval no. XHEC-F-NSFC-2018–124), and also followed the policies of the National Ministry of Health.

### In vivo animal experiments

The targeted tumor accumulation, multimode CT/PA/thermal images, antitumor efficacy assays (including tumor elimination, recurrence suppression, survival ratio and time), systemic toxicity, blood biochemistry index, tissue distribution, biodegradation, and metabolism were performed for different samples. See additional details in the supplementary material.

## Results and discussion

### Synthesis and characterization of MPTF

**Fig. 2:**
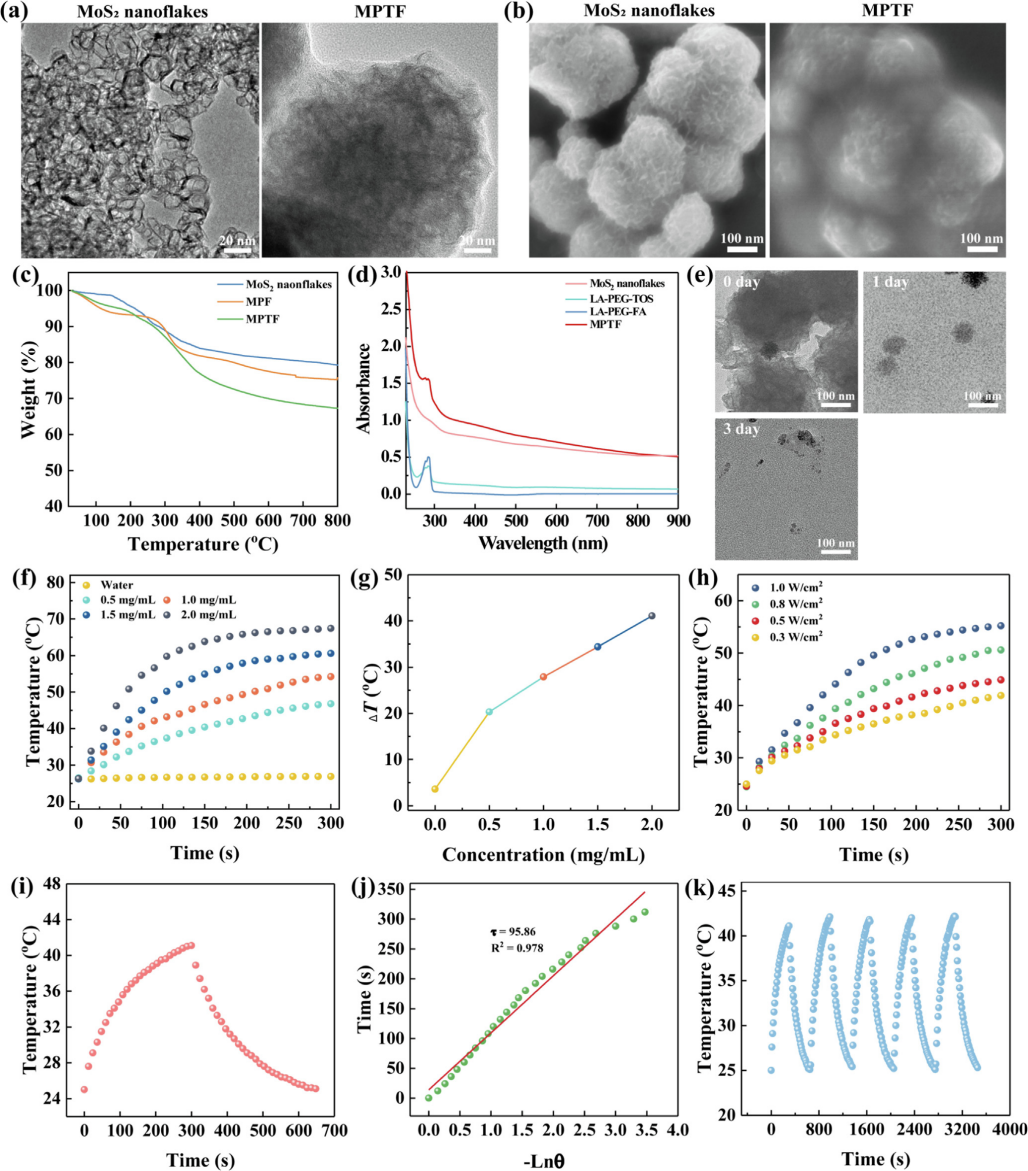
(a) TEM and (b) SEM images of MoS_2_ nanoflakes and MPTF. (c) TGA curves of MoS_2_ nanoflakes, MPF and MPTF. (d) UV–vis-NIR spectra of MoS_2_ nanoflakes, LA-PEG-TOS, LA-PEG-FA and MPTF. (e) TEM images of MPTF after degradation at 0, 1 and 3 days. (f) Temperature elevation and (g) temperature gradient (Δ*T*) of water and the aqueous solution of MPTF at different concentrations under NIR irradiation (808 nm, 1.0 W/cm^2^) for 300 s. (h) Temperature elevation of the aqueous solution of MPTF (1.0 mg/mL) under NIR irradiation (808 nm) with different laser powers for 300 s. (i) Plot of the temperature *vs* time for MPTF (1.0 mg/mL) during NIR irradiation (808 nm, 0.3 W/cm^2^) and cooling (laser off) stages. The laser was closed after irradiation for 300 s. (j) Plot of the cooling time *vs* -lnθ. (k) Temperature plot of the aqueous solution of MPTF (1.0 mg/mL) under NIR irradiation (808 nm, 0.3 W/cm^2^) for five cycles (laser on for 300 s and laser off in each cycle).

The biodegradable MoS_2_ nanoflakes were first synthesized by hydrothermal approach according to our previous work [43]. Then, the targeted ligand FA and anticancer drug α-TOS were conjugated to PEGylated lipoic acid (PEG-LA) by EDC chemistry [44], respectively. According to NMR integration [44], the FA and α-TOS number in each PEG chain were calculated to be 0.83 and 0.95, respectively (Fig. S1). By conversion, the mass contents of FA and α-TOS in each PEG-FA and PEG-TOS chain were 0.181 mg/mg and 0.210 mg/mg, respectively. Finally, these functional PEG chains of LA-PEG-FA and LA-PEG-TOS were modified onto the MoS_2_ nanoflakes *via* disulfide bond to form the MPTF. The micrographs and dimensions of MoS_2_ nanoflakes and MPTF were micro-observed by transmission electron microscopy (TEM) and scanning electron microscopy (SEM) imaging (Fig. 2a,b). Compared to MoS_2_ nanoflakes with a thickness of about 25 nm, the MPTF did not show significant morphology change and was tightly bound to form closely packed spherical particles with a size of around 155 nm [43]. The Mo atoms insert into S atoms stacked in multiple layers to form a prismatic structure. In the process of hydrothermal reaction, prismatic MoS_2_ undergoes directional nuclear aggregation under high temperature and high pressure, that is, a spherical morphology similar to nanoflowers is obtained. Furthermore, the hydrodynamic sizes of MoS_2_ nanoflakes and MPTF were measured using dynamic light scattering (DLS). After functional PEG chains modification of MoS_2_ nanoflakes, the hydrodynamic size of MPTF decreased obviously from 733.7 nm to 209.8 nm (Fig. S2), revealing that the MPTF present better water dispersibility and colloidal stability than the pristine MoS_2_ nanoflakes. Notably, the hydrodynamic size measured by DLS is bigger than that measured by TEM, which is ascribed to the fact that TEM measures the single nanoflakes in a dry state, while DLS measures the clustered nanoflakes in aqueous solution. Due to the functional PEG chains modification, the surface potential of MPTF was higher than that of MoS_2_ nanoflakes (Fig. S3). These results demonstrate that the functional PEG chains modification makes the MPTF more suitable for biomedical applications. Moreover, the quantitative analysis of functional PEG chains within MPTF was performed by thermal gravimetric analysis (TGA) (Fig. 2c). The weight loss of MoS_2_ nanoflakes, MPF, and MPTF was 20.7%, 24.8% and 32.8%, respectively. Through calculation, the percentage of PEG-FA and PEG-TOS chains modified onto the MoS_2_ nanoflakes was 4.6% and 10.6%, respectively. Further combining the results of NMR, the FA and α-TOS amount conjugated onto MPTF was calculated to be 8.4 μg/mg and 22.3 μg/mg, respectively. Likewise, the final Mo content within the MPTF was detected by inductively coupled plasma-optical emission spectroscopy (ICP-OES) to be 538 μg/mg. By the UV–vis spectrum characterization (Fig. 2d), both of MoS_2_ nanoflakes and MPTF showed an obvious absorption in NIR region from 700 nm to 900 nm. In addition, the biodegradability of nanoplatforms is an extremely important character for biomedical applications to avoid the long-term toxicity *in vivo*. The degradable behavior of MPTF under physiological condition of pH 7.4 was observed by TEM imaging (Fig. 2e), and the small polymer fragments remained will be metabolized from the kidney. For the degradation mechanism, some reported work has provided the explanation that the Mo(IV) from MoS_2_ nanoflakes could be oxidized into water-soluble Mo(VI) oxide species (e.g., MoO_4_^2−^) in the presence of OH^−^ [45], [46], [47], [48].

### Photothermal properties

The strong NIR absorption behavior renders the MPTF with photothermal property under NIR irradiation. Next, we studied the photothermal conversion capacity of MPTF with different concentrations under 808 nm laser (1 W/cm^2^) irradiation (Fig. 2f, g). Clearly, after laser irradiation for 5 min, the temperature of water only changed slightly (<1.5 °C). As a comparison, the temperature of MPTF solution significantly increased with the concentration, and the highest temperature change (Δ*T*) reached 41.1 °C at the concentration of 2.0 mg/mL. According to the regulations of ANSI, the MPE of 808 nm laser irradiation for normal skin is 0.33 W/cm^2^ [28]. Therefore, the photothermal behavior of MPTF was further explored under 808 nm laser with different irradiation powers (1.0–0.3 W/cm^2^). Clearly, with the decrease of laser power, the temperature-rise of MPTF was weakened (Fig. 2h and Fig. S4). Under the safe laser irradiation of 0.3 W/cm^2^, the Δ*T* of MPTF decreased to 16.9 °C, which was enough to efficiently ablate cancer cells based on the previous literature [49], [50]. Next, the PCE of MPTF was tested according to our previously reported method [26], [51]. The temperature change curve was obtained when the MPTF solution was heated by laser irradiation for 300 s, and then cooled to room temperature after laser off (Fig. 2i). After that, the τ_s_ was calculated by fitting the cooling time versus –lnθ (Fig. 2j), and the PCE was calculated to be 65.3% which was significantly higher than that of other photothermal agents, for instance, CuS-based (26.7%) [49], Bi-based (30.0%) [52], PDA-based (40.0%) [53], PPY-based (44.7%) [54], and carbon nanodots-based nanoplatforms (53.2%) [55].

Furthermore, the photothermal stability of MPTF was evaluated by the cyclic heating/cooling irradiation upon 808 nm laser (Fig. 2k). It could be seen that MPTF emerges excellent photothermal stability even after multiple NIR irradiation. The superior photothermal stability is a crucial prerequisite for MPTF to obtain higher PCE. These results demonstrate that the MPTF can be used as an excellent photothermal agent for photothermal therapy of tumors upon safe NIR irradiation.

### Biocompatibility and targeting specificity

**Fig. 3:**
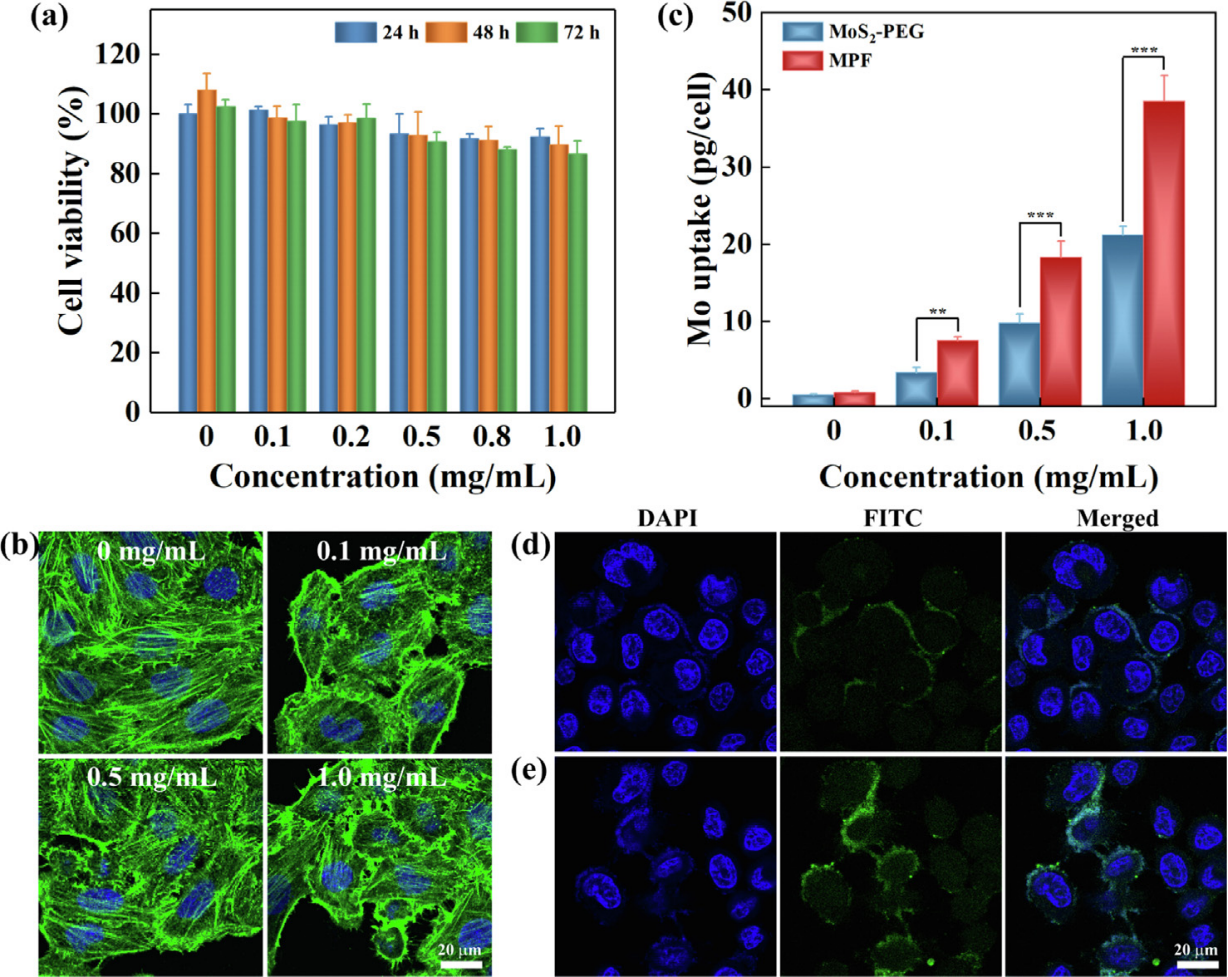
(a) CCK-8 assay of SKoV3 cell viability after exposed to various concentrations of MPF for 24 h, 48 h and 72 h. (b) CLSM images of FITC-phalloidin and DAPI stained SKoV3 cells exposed to various concentrations of MPF for 48 h. (c) Mo uptake in SKoV3 cells exposed to various concentrations of MoS_2_-PEG and MPF for 4 h. CLSM images of SKoV3 cells incubated with (d) MoS_2_-PEG-FI and (e) MPF-FI at the concentration of 0.5 mg/mL for 4 h.

For biomedical applications *in vivo*, the cytocompatibility of MPF needs to be assessed. Through Cell Counting Kit-8 (CCK-8) assay (Fig. 3a), only slight change was revealed in the cell viability treated with MPF at the studied concentration range (0.1–1.0 mg/mL) in comparison to that treated with PBS, and the cell viability was maintained above 86.6% after the co-culture for 24 h, 48 h and 72 h. Furthermore, after treated with MPF for 48 h, the integrity of cell cytoskeleton and nuclei was observed by confocal laser scanning microscopy (CLSM) imaging (Fig. 3b). Apparently, the cell cytoskeleton (green) and nuclei (blue) did not show significant change, similar to the cell treated with PBS. These results imply that the MPF exhibits good biocompatibility.

The specific cellular uptake for the MPF was examined quantitatively by ICP-OES analysis of Mo element content (Fig. 3c). The Mo uptake in the cell for MPF was about 1.8–2.2 times higher than that for MoS_2_-PEG at different concentrations. Furthermore, to visualize the cellular internalization, the green fluorescent molecule of FITC was conjugated onto the MoS_2_-PEG and MPF. By CLSM imaging (Fig. 3d,e), the higher fluorescence intensity was observed in the cells treated with MPF-FI when compared to that treated with MoS_2_-PEG-FI. Overall, the MPF comes up with enhanced cellular internalization of SKoV3 cells by FA-mediated specific targeting.

### Selective anticancer activity and photothermal ablation of ovarian cancer cells

**Fig. 4:**
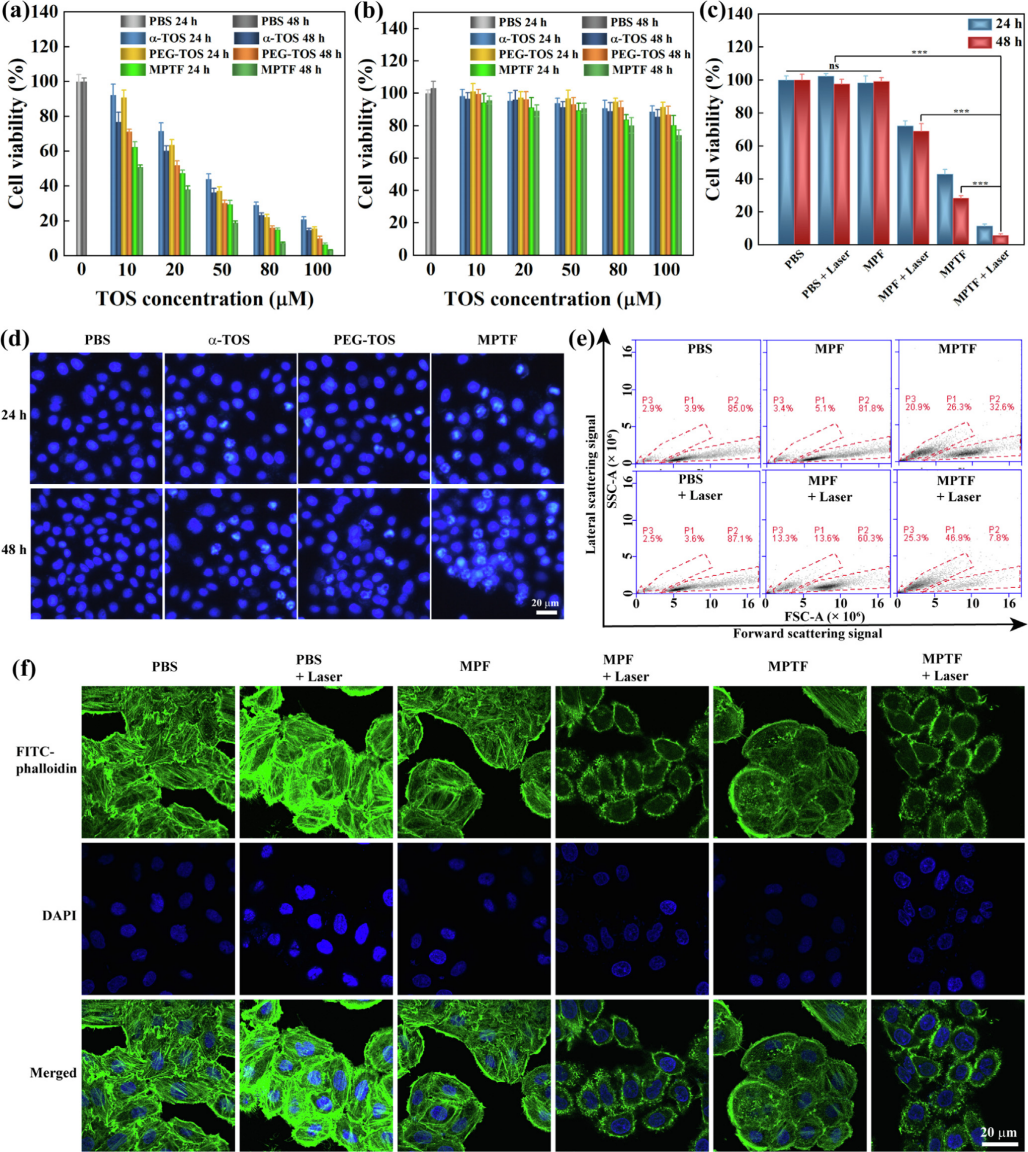
Viability assay of (a) SKoV3 cells and (b) Lec1 cells treated with PBS, α-TOS, PEG-TOS, MPTF with different TOS concentrations of 10–100 μM (equivalent to α-TOS concentrations of 5.3–53.2 μg/mL, PEG-TOS concentrations of 25.3–250.3 μg/mL, and MPTF concentrations of 0.238–2.38 mg/mL) for 24 h and 48 h. (c) CCK-8 viability of SKoV3 cells after different treatments at the concentration of 0.2 mg/mL (equivalent to α-TOS concentration of 8.4 μM) with or without NIR irradiation (808 nm, 0.3 W/cm^2^) for 10 min. (d) Fluorescence microscopic images of SKoV3 cells (nuclei stained by Hoechst 33342) treated with PBS, α-TOS, PEG-TOS, MPTF at the TOS concentration of 30 μM for 24 h and 48 h. (e) Flow cytometric assay of SKoV3 cells after different treatments. The x and y axes reflect the particle size and the internal complexity of particle, respectively. P1, P2 and P3 represent the apoptotic bodies, the cells with normal structure, and the cell fragments, respectively. (f) CLSM images of SKoV3 cells (cytoskeleton stained by FITC-phalloidin and nuclei stained by DAPI) after different treatments.

All of first, the selective chemotherapy effect of MPTF for SKoV3 cells was evaluated by CCK8 assay (Fig. 4a). The cell viability in MPTF and PEG-TOS groups was lower than that in free α-TOS group at the same concentration and co-culture time. Moreover, the half maximal inhibitory concentrations (IC_50_s) of α-TOS, PEG-TOS and MPTF for SKoV3 cells at 24 h and 48 h were calculated (Fig. S5 and Table S2), and the order of IC_50_ is as follows: α-TOS 24 h (42.3 μM) > PEG-TOS 24 h (35.4 μM) > α-TOS 48 h (32.7 μM) > PEG-TOS 48 h (22.5 μM) > MPTF 24 h (17.5 μM) > MPTF 48 h (9.8 μM). These results indicate that the conjugation of PEG-TOS and PEG-FA onto the MoS_2_ nanoflakes can improve greatly the anticancer activity of α-TOS, owing to the afforded water dispersibility and FA-mediated targeting specificity.

The mechanisms of anticancer activity of MPTF was further disclosed by the cell nuclei staining with Hoechst 33,342 (Fig. 4d and Fig. S6). The typical apoptotic morphologic change of hyperchromatic nuclei (punctate blue fluorescence) occurred in the cells treated with α-TOS, PEG-TOS, and MPTF, implying that α-TOS is able to induce cell apoptosis by prolonging cell cycle arrest. Meanwhile, compared to α-TOS group, a more obvious chromatic condensation was shown in PEG-TOS and MPTF groups. Also, for commonly used anticancer drugs, they indiscriminately kill cancer and normal cells. What exciting is that MPTF in the studied concentrations did not show obviously toxicity to normal ovarian (Lec1) cells (Fig. 4b), attributing to the high esterase activity, compartmental integrity and lack of annexin V binding in normal cells [56], [57]. Overall, the selective chemotherapy of MPTF is a very important feature to avoid the side effects of systemic administration.

Next, the combined photothermal-selective chemotherapy of MPTF for SKoV3 cells under safe NIR irradiation (808 nm, 0.3 W/cm^2^) was further explored by CCK-8 assay (Fig. 4c). The higher cancer cells inhibition efficacy was observed in MPTF + Laser group (representing combined therapy) compared to that in MPF + Laser group (representing single photothermal ablation) and MPTF group (representing single chemotherapy), respectively. Specifically, after co-culture for 48 h, the cell viability in MPTF + Laser group decreased to 5.7%, which was apparently lower than that in MPF + Laser (68.9%, p < 0.001) and MPTF groups (28.3%, p < 0.001), respectively. Similarly, the flow cytometry of cells with different treatments also revealed that the combined therapy could significantly damage the cancer cells by either apoptotic or necrotic pathways than single photothermal ablation or chemotherapy (Fig. 4e). To further reveal the synergistic effect of photothermal-selective chemotherapy, the combination index (CI) was calculated according to the literature [58]. The CI was calculated to be 0.82 (<1), implying the synergistic effect of photothermal-selective chemotherapy based on MPTF with NIR laser (Fig. S7). It can be found that the combinational therapy in MPTF with safe NIR irradiation present an enhanced significantly therapeutic effect when compared to the photothermal therapy or chemotherapy alone.

Furthermore, the mechanism of combined therapy for SKoV3 cells was illustrated by the visualization of cell cytoskeleton and nuclei using CLSM images (Fig. 4f). Clearly, the cells exhibited intact cytoskeleton and nuclei in control groups. For comparison, photothermal therapy in MPF + Laser group can ablate the cancer cells by the disruption of actin stress fibers, while chemotherapy in MPTF group was to inhibit the growth of cancer cells due to the enlarged cell by chromatic condensation even if the cytoskeleton remains relatively intact. The MPTF under safe NIR irradiation display the combined therapeutic efficacy to enhance anticancer effect.

### Targeted accumulation and multimode CT/PA/Thermal images of tumor

**Fig. 5:**
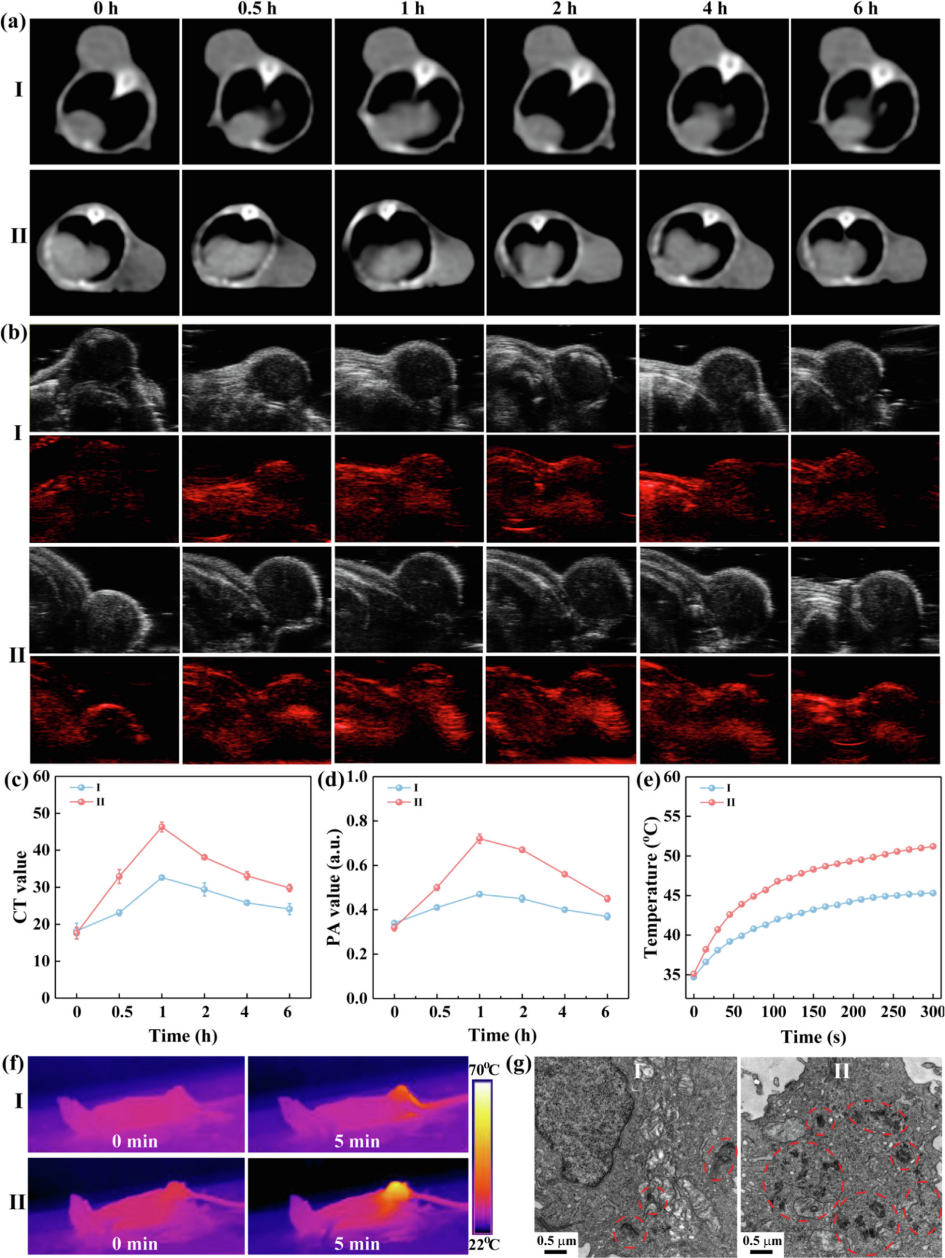
(a) CT and (b) PA images and corresponding (c) CT and (d) PA values of SKoV3 tumors in nude mice at different time points after i.v. injection of (I) MPT and (II) MPTF. (e) Temperature profiles and (f) thermal images of SKoV3 tumors in nude mice under NIR irradiation after i.v. injection of I and II. (g) Bio-TEM images of tumor tissues obtained at 1 h after i.v. injection of I and II.

For theranositc application *in vivo*, we established the subcutaneous xenograft of SKoV3 in nude mice as ovarian carcinoma model. The multimode images ability of theranostic nanoplatforms is critical for the accurate diagnosis, tumor localization, post-treatment and therapeutic outcome monitoring [44], [59]. After intravenous injection (i.v.) of MPT and MPTF (0.2 mL, 4 mg/mL), the CT/PA images (Fig. 5a, b) and corresponding signal intensity (Fig. 5c, d) were obtained at different time intervals. The CT/PA signal values of tumor increased with postinjection time and reached the peak value at 1 h. More importantly, at the peak time of 1 h, the tumor CT and PA signal values in MPTF group were 1.52 times and 1.63 times higher than that in MPT group, respectively. These results indicate that the enhanced tumor accumulation of MPTF is achieved by specific targeting, and the highest concentration can be reached in the tumor region at 1 h postinjection. Moreover, at peak time, the Bio-TEM images of tumor tissue were obtained (Fig. 5g and Fig. S8), and the higher tumor accumulation of MPTF was observed than that of MPT. This result further implies that the FA modification renders the MPTF with the specific accumulation in tumor region *in vivo*.

Additionally, in MPT and MPTF groups, the 808 nm laser irradiation (0.3 W/cm^2^) was performed in the tumor at 1 h postinjection to obtain the thermal images (Fig. 5e, f). Excitingly, the tumor temperature in MPTF group increased rapidly to around 51.2 °C, much higher than that in MPT group (45.3 °C). This is due to the fact that the MPTF can accumulate in tumor region more effectively by FA-mediated targeting specificity *in vivo*. According to the previous reports [49], [50], cancer cells can be damaged irreversibly at the temperature of about 45–50 °C. Therefore, the elevated temperature of tumor in MPTF group under safe NIR irradiation (0.3 W/cm^2^) is high enough to kill cancer cells. Taken together, the MPTF can be used as a promising contrast agent for targeted multimode CT/PA/thermal images, which is beneficial to improve the diagnostic sensitivity and localization accuracy of tumors. Likewise, the targeted multimode images thus subsequently guide the combined therapy of tumors.

### Efficient therapy and recurrence suppression of tumor under safe NIR irradiation

**Fig. 6:**
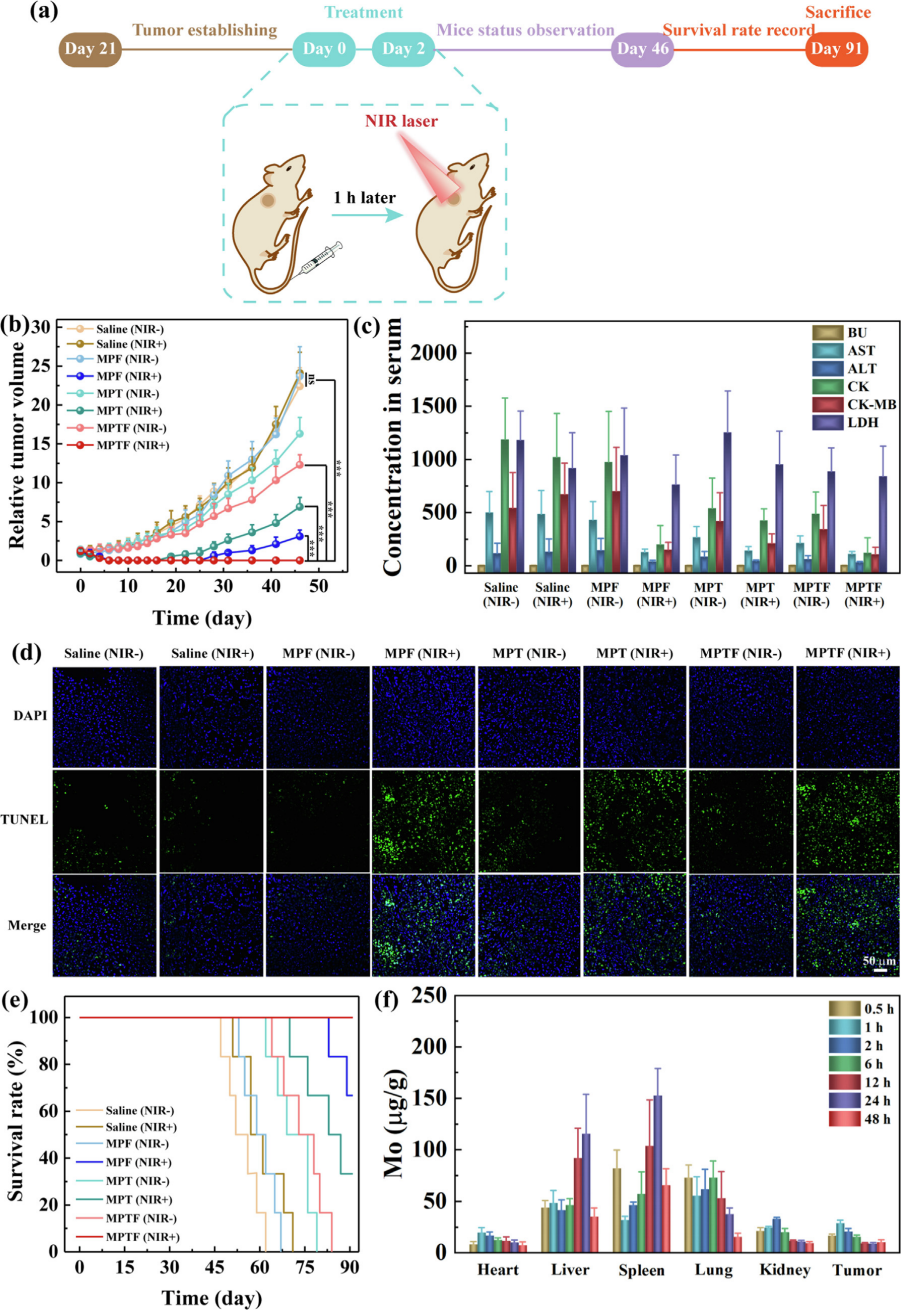
(a) Schematic illustration and timeline of the combined therapy of MPTF by systemic injection under safe NIR irradiation. (b) Relative tumor variation of the mice as a function of time in different treatment groups. (c) The blood biochemical parameters *in vivo* in different treatment groups. (d) TUNEL staining of tumor sections in different treatment groups. (e) Survival rate of the mice as a function of time in different treatment groups. (f) Biodistribution of Mo element in different organs and tumors at different times postinjection of MPTF.

Due to the insufficient tumor accumulation or/and low PCE of some photothermal agents, the NIR irradiation with excessive power (more than the MPE of 808 nm laser, 0.33 W/cm^2^) was often conducted to obtain efficient photothermal therapy for tumor ablation in previous some work (Table S1). The NIR irradiation with excessive power is permitted for the fundamental research of photothermal therapy, while the safety of NIR irradiation must be considered in clinical transformation and practice. Based on these above results of combined therapy *in vitro* and targeted multimode images *in vivo*, we believe that an excellent antitumor efficacy with low side effects through the clinical concept can realize under safe irradiation (808 nm, 0.3 W/cm^2^). The treatment timeline of the nude mice bearing SKoV3 tumor in MPTF group was given in Fig. 6a. It is encouraging that the tumors in MPTF (NIR+) group were eliminated thoroughly on day 6 after treatment, and no tumor recurrence was observed during 46 days, or even 91 days (Fig. 6b and Fig. S9). As a contrast, the recurrence of tumors occurred at 19 days in MPT (NIR+) group after treatment, suggesting that the enhanced tumor accumulation is able to improve tumor therapeutic efficacy. Moreover, in MPF (NIR+) group, the tumor recurrence occurred at 28 days after treatment, mainly because of single photothermal therapy with low NIR irradiation. The tumor growth in MPTF (NIR−) group was inhibited to a certain extent due to the limitation of single chemotherapy.

Furthermore, the TUNEL staining of tumor slices was carried out to evaluate the combined therapy effect (Fig. 6d). The quantitative analysis of cell apoptosis rate in tumors for different groups was as follows (Fig. S10): MPTF (NIR+) (87.5%) > MPF (NIR+) (66.9%) > MPT (NIR+) (53.8%) > MPTF (NIR−) (29.3%) > MPT (NIR−) (18.4%) > MPF (NIR−) (7.5%) ≈ Saline (NIR−) (8.7%) ≈ Saline (NIR+) (7.2%). All the results demonstrate that compared with photothermal therapy or chemotherapy alone, the combined photothermal-selective chemotherapy exerts high potency in tumor treatment that is expected to clear the residual cancer cells and significantly inhibit tumor recurrence.

### Systemic toxicity, biodistribution and biodegradation *in vivo*

For ideal therapeutic strategy, apart from efficient tumor elimination, the negligible side effect is also necessary. The average weight of mice in each group was measured, and no obvious body weight loss was presented after treatment for all groups (Fig. S11). Likewise, to furtherly detect the systemic toxicity and long-term side effect, the main organs of mice (heart, liver, spleen, lung and kidney) were removed and H&E stained on day 46 in different treatment groups (Fig. S12). These organs in different treatment groups did not show any necrosis region, similar to Saline (NIR−) group. More importantly, to further evaluate the clinical translation potential of the theranostic nanoplatforms and strategy, the major blood biochemistry parameters, containing blood urea (BU), aspartate aminotransferase (AST), alanine aminotransferase (ALT), creatine kinase (CK), CK-muscle/brain (CK-MB) and lactate dehydrogenase (LDH), were recorded in different groups (Fig. 6c). These parameters of mice in different groups are not significantly difference. Despite this, the indexes of AST, ALT, CK and CK-MB have the highest levels in control groups (Saline (NIR−), Saline (NIR+) and MPF (NIR−) groups), while that emerge the low values in the treatment group of MPTF (NIR+), indicating that the burden of large tumor in control groups lead to multi-organs failure and the effective treatment in MPTF (NIR+) group can protect the internal organs. These results imply that the designed theranostic nanoplatforms and the utilization of photothermal-selective chemotherapy strategy exhibit good biosafety and have no adverse effects for next clinical translation.

In addition, the mice survival, the ultimate goal of all treatments, was monitored during 91 days (Fig. 6e). The mice in the control groups were all dead from day 62 to day 71. While the survival rate in the groups with single photothermal therapy, single chemotherapy or non-targeted combined therapy was as follow: MPF (NIR+) (66.7% on day 91), MPT (NIR+) (33.3% on day 91), MPTF (NIR−) (0% on day 84), and MPT (NIR−) group (0% on day 78). It is worth mentioning that 100% survival rate occurred in MPTF (NIR+) group after 91 days. These results confirm that the treatment of MPTF under safe NIR irradiation not only achieve superior tumor elimination with no recurrence but also display negligible side effect, leading to sufficiently prolonged survival life-span of mice.

Finally, the metabolic pathway of MPTF in the mice was tracked. After i.v. injection, the biodistribution of Mo element in major organs and tumors at different time points was measured by ICP-OES (Fig. 6f). The highest amount of Mo element was accumulated in the tumor tissue at 1 h post-injection, and the value was maintained at a relatively high level until 48 h, due to the EPR and active targeting effect. In the liver and spleen, the Mo elements were gradually accumulated at 24 h postinjection and then metabolized after 24 h, implying that the MPTF *in vivo* can be cleared by the reticuloendothelial system organs. Additionally, beginning on Day 3, Mo shown a decrease in the spleen and an increase in the liver (Fig. S13), which may result from the degradation of MPTF and their further translocating *via* the splenic vein to the hepatic portal vein and finally being utilized in molybdenum enzymes, consistent with the previous work [47]. Likewise, the excretion of MPTF *in vivo* after injection for one week was explored (Fig. S14). Clearly, the Mo element was detected from the urines of mice on the first day of injection. As a comparison, the Mo element in feces was negligible.

## Conclusion

In summary, for the first time, a safe and efficient platform of α-TOS married 2D MoS_2_ was successfully developed for cooperative CT/PA/thermal imaging-guided photothermal-selective chemotherapy of ovarian cancer under safe NIR irradiation. The created MPTF displaying valuable biodegradability, high PCE, excellent photothermal stability, good biocompatibility, specific targeting, selective anticancer activity and enhanced tumor accumulation can be exploited for precise images and enhanced tumor therapy with negligible side effects. Notably, the PCE of 65.3% of the platform under safe NIR irradiation is much higher than that of other photothermal materials reported elsewhere. Likewise, the combined photothermal-selective chemotherapy is capable of eliminating completely the solid tumor by photothermal therapy under safe NIR irradiation, and then killing the residual cancer cells by chemotherapy for prevented tumor recurrence. More importantly, the excellent efficacy and safety benefits *in vivo* lead to the prominent survival rate of tumor-bearing mice to reach 100% over 91 days. For the weak patients with advanced ovarian cancer, the photothermal-selective chemotherapy is an ideal combinational regimen to replace clinical surgery and chemotherapy to avoid large risks of surgery and severe side effects of systemic chemotherapy. Based on the successful evaluation *in vivo*, the designed theranostic platform would provide not only a promising candidate in preclinical nanomedicine libraries but also a blueprint for new generation of nanomedicine in potential clinical applications.

## Compliance with Ethics Requirements

*All Institutional and National Guidelines for the care and use of animals were followed.*

*Animal study Ethics statement: All animal experiments involving animals were conducted according to the ethical policies and procedures approved by the Ethical Committee of Shanghai XinHua Hospital (Approval no. XHEC-F-NSFC-2018-124), and also followed the policies of the National Ministry of Health.*

## CRediT authorship contribution statement

**Xin Li:** Conceptualization, Methodology, Software, Data curation, Writing – original draft. **Lingdan Kong:** Data curation, Formal analysis, Writing – original draft. **Wei Hu:** Methodology, Software, Data curation. **Changchang Zhang:** Methodology, Software, Data curation. **Andrij Pich:** Methodology, Software, Data curation. **Xiangyang Shi:** Supervision, Resources, Funding acquisition, Writing – review & editing. **Xipeng Wang:** Supervision, Resources, Funding acquisition, Writing – review & editing. **Lingxi Xing:** Conceptualization, Supervision, Resources, Funding acquisition, Project administration, Writing – review & editing.

## Declaration of Competing Interest

*The authors declare that they have no known competing financial interests or personal relationships that could have appeared to influence the work reported in this paper*.
