# Supplementary material for: Safe and efficient 2D molybdenum disulfide platform for cooperative imaging-guided photothermal-selective chemotherapy: A preclinical study
Source: J Adv Res. 2021 Aug 11;37:255–66. doi: 10.1016/j.jare.2021.08.004 (PMC9039738; doi:10.1016/j.jare.2021.08.004)
Supplement: Supplementary data 1 [file mmc1.docx]

Supplementary Material

Safe and efficient 2D molybdenum disulfide platform for cooperative imaging-guided photothermal-selective chemotherapy: A preclinical study

**Additional experimental details:**

*Materials:* Ammonium tetrathiomolybdate ((NH_4_)_2_MoS_4_), hydrazinium hydroxide ((N_2_H_4_)·H_2_O, 98%) and lipoic acid (LA, 98%) were purchased from J&K China Chemical Co., Ltd. (Beijing, China). t-butyloxy carbonyl protected polyethylene glycol with amine group (BocNH-PEG-NH_2_, Mw = 2000) was obtained from Shanghai Yanyi Biotechnology Corporation (Shanghai, China). 1-ethyl-3-(3-dimethylaminopropyl) carbodiimide hydrochloride (EDC), N-hydroxysuccinimide (NHS), dimethylsulfoxide (DMSO), hydrochloric acid (HCl), folic acid (FA), fluorescein isothiocyanate (FITC), paraformaldehyde, Triton® X-100 and bovine serum albumin (BSA) were supplied from Sigma-Aldrich (St. Louis, MO). α-tocopheryl succinate (α-TOS) was acquired from Hubei Hengshuo Chemical CO., Ltd. (Wuhan, China). SKoV3 cells (human ovarian cancer cell line) were provided from Institute of Biochemistry and Cell Biology, the Chinese Academy of Sciences (Shanghai, China). Dulbecco's Modified Eagle Medium (DMEM) and fetal bovine serum (FBS) were purchased from Hangzhou Jinuo Biomedical Technology (Hangzhou, China). Penicillin-streptomycin and trypsin were obtained from HyClone Lab., Inc. (Logan, UT). Cell Counting Kit-8 (CCK-8) was supplied from 7Sea Biotech. Co., Ltd. (Shanghai, China). 4',6-diamidino-2-phenylindole (DAPI) and Hoechst 33342 were acquired from Bestbio Co., Ltd. (Shanghai, China). FITC-phalloidin was provided from Invitrogen (Carlsbad, CA). Regenerated cellulose dialysis membranes with a molecular weight cutoff (MWCO) of either 1000 or 8000-14000 were obtained from Fisher Scientific (Pittsburgh, PA). Water used in all experiments was purified using a Milli-Q Plus 185 water purification system (Millipore, Bedford, MA) with a resistivity higher than 18.2 MΩ·cm.

*Synthesis of MoS2-PEG-FI and MPF-FI:* To visualize the targeted cellular internalization, the FITC conjugated PEG was synthesized. In brief, LA-PEG-FA (100 mg) dissolved in 2 mL DMSO was reacted with FITC (19 mg, in 2 mL DMSO) under vigorous stirring for 24 h in the dark. Subsequently, the solution was dialyzed using dialysis membrane (MWCO = 1000) and freeze-dried to obtain LA-PEG-FA-FI. After that, the MoS_2_ nanoflakes (50 mg) dispersed in 5 mL water were reacted with LA-PEG-FA-FI (250 mg, in 3 mL water) under stirring for 12 h. The reaction mixture was dialyzed using dialysis membrane (MWCO = 8000-14000) and freeze-dried to obtain the product of MoS_2_-PEG-FA-FI (MPF-FI for short). Moreover, the LA-PEG-FI without FA modification and MoS_2_-PEG-FI were also prepared as control samples under same experimental condition.

*Characterization techniques:* ^1^H NMR spectra were collected on a Bruker AV400 nuclear magnetic resonance spectrometer using D_2_O as solvent. Transmission electron microscopy (TEM) imaging was performed on a JEOL 2010F analytical electron microscope (JEOL, Tokyo, Japan) operating at a voltage of 200 kV. The samples were prepared by depositing a diluted particle suspension onto carbon-coated copper grid and air dried before imaging. Field emission scanning electron microscopy (FESEM) was carried out using a Hitachi scanning electron microscope (Tokyo, Japan) at an operating voltage of 5 kV. The samples were prepared by depositing a diluted particle suspension onto aluminum foil and air dried before imaging, subsequently the samples were sputter coated with a thin film of Au with a thickness of 10 nm. UV-vis spectra was recorded using a Lambda 25 UV-vis spectrophotometer (PerkinElmer, Boston, MA). Thermal gravimetric analysis (TGA) was performed using a TG 209 F1 thermal gravimetric analyzer (NETZSCH Instruments Co., Ltd., Selb/Bavaria, Germany) at a heating rate of 20 ^o^C/min under a flowing N_2_ atmosphere. Dynamic light scattering (DLS) measurements were conducted using a Malvern Zetasizer Nano ZS model ZEN3600 (Worcestershire, UK) equipped with a standard 633 nm laser at room temperature. Leeman Prodigy inductively coupled plasma-optical emission spectroscopy (ICP-OES, Hudson, NH) was performed to analyze the Mo concentration in aqueous solution.

*Photothermal properties:* The photothermal conversion was evaluated by recording the temperature change of MPTF exposed to NIR irradiation of 808 nm laser using the laser device (Shanghai Xilong Optoelectronics Technology Co. Ltd., Shanghai, China). Typically, the aqueous solution of MPTF (0.3 mL) with different concentrations (0-2.0 mg/mL) was placed in 0.5 mL Eppendorf tube and irradiated by 808 nm laser (1.0 W/cm^2^) for 300 s, and the temperature of the solution was tracked every 15 s using a thermocouple probe (Shenzhen Everbest Machinery Industry Co., Ltd., Shenzhen, China). Besides, the temperature change of MPTF (0.3 mL, 1.0 mg/mL) exposed to 808 nm laser with different power densities (0.3-1.0 W/cm^2^) was recorded in the same way.

Quantitative measurement of the photothermal conversion efficiency (*η*_PCE_) of MPTF was carried out. The aqueous solution of MPTF (0.3 mL, 1.0 mg/mL) was placed in 0.5 mL Eppendorf tube and irradiated by 808 nm laser (0.3 W/cm^2^), and the laser was turned off after irradiation for 300 s. The *η*_PCE_ was calculated according to the following Eq. (1): [1, 2]

$\eta_{\mathrm{PCE}}= \frac{hS\left( T_{max}-T_{sur} \right)-Q_{s}}{I(1-{10}^{{-A}_{\lambda}})}$ (1)

Where *h* is the heat transfer coefficient, *S* the surface area of the sample cuvette, *T_max_* the steady-state temperature, *T_sur_* the temperature of the surroundings, *Q_s_* the heat associated with the light absorbance of the solution, *I* the incident laser power, and *A_λ_* the absorbance at a wavelength of 808 nm.

Moreover, to test the photothermal conversion stability, the aqueous solution of MPTF (0.3 mL, 1.0 mg/mL) was subjected to five cycles of 808 nm laser (0.3 W/cm^2^) irradiation/cooling down process.

*Biocompatibility and targeting specificity in vitro:* Cell Counting Kit-8 (CCK-8) assay was used to evaluate the cytotoxicity of MPF at different concentrations. SKoV3 cells were seeded into a 96-well plate at a density of 1.0 × 10^4^ cells/well with fresh medium (DMEM concluding 10% FBS and 1% penicillin-streptomycin) the day before the experiment. Then the medium in each well was replaced with 0.1 mL fresh medium containing phosphate buffered saline (PBS) or MPF (10 μL) with the final concentration ranging (0.1-1.0 mg/mL). The cells were incubated at 37 ^o^C and 5% CO2 for another 24 h, 48 h and 72 h. Then the cells were washed 3 times with PBS, and the CCK8 (10 μL) was added into each well with fresh medium (100 μL) and then the cells were incubated continuously for another 3 h. Finally, the absorbance at 450 nm in each well was recorded using a Multiskan MK3 ELISA reader (Thermo Scientific, Waltham, MA).

Furthermore, the morphology of cytoskeleton and cell nuclei of SKoV3 cells after co-culture with MPF was observed using a Carl Zeiss LSM 700 confocal laser scanning microscopy (CLSM, Jena, Germany). The coverslips were placed in 12-well plate and soaked using DMEM for 24 h. The SKoV3 cells were seeded into each well with coverslip at a density of 2.0 × 10^5^ cells/well and incubated overnight, then the cells were incubated with 0.5 mL fresh medium containing PBS or MPF (50 μL) with different concentrations (0.1-1.0 mg/mL) for another 48 h. The culture medium was removed and each well was washed 3 times with PBS, and the cells were fixed with fresh paraformaldehyde (4.0%) for 30 min at room temperature. Then, the cells were permeabilized with 0.1% Triton X-100 in PBS for 10 min and blocked with 1% BSA in PBS for 30 min. The cells were subjected to F-actin staining with FITC-phalloidin for 30 min and DAPI for 5 min in the dark before CLSM imaging. After each step of processing, the cells were washed 3 times with PBS.

To investigate the FA-mediated specific targeting of cellular internalization, the SKoV3 cells were incubated with MoS_2_-PEG and MPF respectively at different concentrations. The cells were seeded at a density of 4 × 10^6^ cells in a 25 cm^2^ culture flask with fresh medium. After overnight culture, the medium was replaced with 3 mL fresh medium containing PBS, MoS_2_-PEG or MPF (300 μL) at different concentrations (0.1-1.0 mg/mL), and the cells were incubated for another 6 h. Subsequently, the cells were washed for 3 times with PBS, lifted with trypsinization, centrifuged, and resuspended in PBS. The cells were counted and lysed using an aqua regia solution (1 mL) to digest both the cells and samples. Each sample was diluted with 2 mL of water before quantification of the Mo concentration using ICP-OES.

Additionally, the visualization of specifically targeted uptake was performed using CLSM imaging. In brief, the adherent SKoV3 cells in 12-well plate with coverslip cultured as described above were incubated with 0.5 mL fresh medium containing MoS_2_-PEG-FI or MPF-FI (50 μL) at the concentration of 0.5 mg/mL for 6 h. Then, the DAPI staining and CLSM imaging of cells were carried out to observe cellular uptake according to protocols described above.

*Selective anticancer activity and photothermal ablation in vitro:* CCK-8 assay was used to evaluate the anticancer activity of free α-TOS, PEG-TOS and MPTF at different concentrations and treatment times. The adherent SKoV3 cells in 96-well plate cultured as described above were incubated with 0.1 mL fresh medium containing PBS, α-TOS, PEG-TOS, or MPTF (10 μL) with the final TOS concentration ranging (10-100 μM) for 24 h and 48 h. After that, CCK-8 assay was performed to measure the cell viability according to protocols described above. To test the selective anticancer activity, the toxicity of α-TOS, PEG-TOS, and MPTF for Lec1 cells (Chinese hamster ovary cells) was further evaluated by CCK-8 assay in the same way. The half maximal inhibitory concentration (IC_50_) in different groups was calculated by GraphPad Prism software (GraphPad Software Inc., San Diego. CA).

Moreover, the cell apoptosis assay was carried out using CLSM imaging. Typically, the adherent SKoV3 cells in 12-well plate with coverslips cultured as described above were incubated with 0.5 mL fresh medium containing α-TOS, PEG-TOS, or MPTF (50 μL) at the TOS concentration of 30 μM for 24 h or 48 h. Subsequently, the culture medium was removed and each well was washed 3 times with PBS, and the cells were fixed with glutaraldehyde (2.5%) for 15 min at 4 ^o^C. Then, the cells were washed 3 times with PBS and counterstained with Hoechst 33342 (1 μg/mL) for 5 min at room temperature before CLSM imaging.

In addition, to evaluate the combined therapeutic efficacy, the adherent SKoV3 cells in 96-well plate cultured as described above were incubated with 0.1 mL DMEM containing PBS, MPF or MPTF (10 μL, 0.2 mg/mL) for 24 h or 48 h, and then the cells were treated with or without the irradiation of 808 nm laser (0.3 W/cm^2^) for 10 min. After that, CCK-8 assay was carried out to determine the cell viability according to protocols described above. Furthermore, the cell viability after different treatments was analyzed by flow cytometric assay. Briefly, the adherent SKoV3 cells in 24-well plate cultured as described above were incubated with 0.5 mL DMEM containing PBS, MPF or MPTF (50 μL, 0.2 mg/mL) for 48 h, and then the cells were treated with or without the irradiation of 808 nm laser (0.3 W/cm^2^) for 10 min. Subsequently, cells were collected and resuspended in binding buffer, incubated with Annexin V-FITC for 15 min, and PI for 5 min at room temperature respectively, and finally detected by Accuri C6 Flow cytometer (Becton Dickinson, New Jersey, USA).

Finally, the morphology of cytoskeleton and cell nuclei was observed using CLSM imaging. The adherent SKoV3 cells in 12-well plate with coverslips cultured as described above were incubated with 0.5 mL fresh medium containing PBS, MPF or MPTF (50 μL, 0.2 mg/mL) for 48 h. Then, the cells were treated with or without the irradiation of 808 nm laser (0.3 W/cm^2^) for 10 min. After that, the FITC-phalloidin and DAPI staining as well as CLSM imaging were carried out to observe cellular morphology according to protocols described above.

*Targeted tumor accumulation and multimode CT/PA/thermal images in vivo:* Animal experiments were performed following the protocols approved by the institutional committee for animal care and the policy of the National Ministry of Health. The 5-week old male nude mice (22-25 g) were purchased from Shanghai Slac Laboratory Animal Center (Shanghai, China). One mouse were subcutaneously injected with 5 × 10^6^ SKoV3 cells in the back, and then the tumor nodule having a volume of 0.9-1.2 cm^3^ at 3 weeks postinjection. After that, the tumor nodule was taken out from mouse, and then evenly cuted into small pieces and transplanted to another new mice. When these tumor volume reached 0.5-0.8 cm^3^, the MPT and MPTF dispersed in 0.2 mL saline (4 mg/mL) were intravenously injected to each mouse via tail vein. We collected the computed tomography (CT) images using a clinical LightSpeed VCT CT imaging system (GE Medical Systems, Milwaukee, WI) with 80 kV, 500 mA, and a slice thickness of 0.625 mm, and photoacoustic (PA) images using VEVO LAZR-X photoacoustic imaging system (Fujifilm VisualSonics, WA, USA) at different time points (0-6 h) post intravenous injection (i.v.), and the corresponding CT/PA values of tumor were quantified, respectively. For thermal images, the tumor region was irradiated by 808 nm laser (0.3 W/cm^2^) for 300s, and then the IR thermographs of mice were collected and the corresponding temperature change in tumor region was recorded using the infrared camera. Moreover, after i.v. injection of MPT and MPTF, tumor tissues of mice was taken at the peak time, and Bio-TEM images were obtained to observe the tumor accumulation of samples.

*Combined therapy and systemic toxicity in vivo:* The mice-bearing ovarian cancer model was established to have a tumor volume of about 0.12 cm^3^, and then randomly divided into 8 groups (n = 6 for each group): Saline (NIR-), Saline (NIR+), MPF (NIR-), MPF (NIR+), MPT (NIR-), MPT (NIR+), MPTF (NIR-) and MPTF (NIR+). On day 0 and day 2, each mouse was tail vein intravenously injected with Saline, MPF, MPT or MPTF (0.2 mL, 4 mg/mL), and then the tumor was treated with or without 808 nm laser irradiation (0.3 W/cm^2^) at 1 h postinjection. At the required time point, the relative tumor volume, body weight and survival rate of all mice were recorded and the pictures of mice were taken by digital camera. The relative tumor volume (*Y*_T_) and survival rate (*η*_SR_) calculated by Eqs. (2) and (3), respectively:

$Y_{T}=V/V_{ini}$, $V=W^{2}\times L/2$ (2)

$\eta_{\mathrm{SR}}=N_{sur}/N_{tot}\times100\%$ (3)

Where *V* and *V_ini_* are the tumor volume after treatment and the initial tumor volume before treatment, *W* and *L* are the width and length of the tumor, *N_sur_* and *N_tot_* are the number of surviving mice and total mice in each group, respectively.

Besides, after different treatments, the tumor tissue of mice in each group was removed on day 2, fixed in 10% formalin, sectioned, and stained using TdT-mediated dUTP Nick-End Labeling (TUNEL) staining. Lastly, the specimens were observed by fluorescence microscope (Carl Zeiss, Axio Vert. A1, Jena, Germany), and the cell apoptosis rate of tumor was quantified. The percentage of TUNEL-positive cells in each sample was determined from five random selected fields. After 91 days, the blood of mice in different groups were collected to detect the biochemistry index, including blood urea (BU, mmol/L), aspartate aminotransferase (AST, U/L), alanine aminotransferase (ALT, U/L), creatine kinase (CK, U/L), CK-muscle/brain (CK-MB, U/L) and lactate dehydrogenase (LDH, U/L), to test the major organ function.

*Histological analysis, biodistribution and biodegradation:* For histological analysis, the major organs at 46 days post-injection in each group were harvested, fixed in 4% paraformaldehyde, embedded in paraffin and then sectioned into slices for hematoxylin and eosin (H&E) staining. For biodistribution study, MPTF were injected into each mouse *via* tail vein (0.2 mL, 4 mg/mL). Subsequently, the mice were anesthetized and sacrificed at different times postinjection, respectively. Then the major organs and tumors in the mice were harvested, weighted, shred and digested by aqua regia for 3 days. Moreover, the urine and faces were daily collected within one week. Finally, the Mo content in all the samples was quantified by ICP-OES.

*Statistical analysis:* One-way analysis of variance (ANOVA) statistical method was performed to evaluate the experimental data. A value of 0.05 was selected as the significance level and the data were indicated with (*) for *p* < 0.05, (**) for *p* < 0.01, and (***) for *p* < 0.001, respectively.


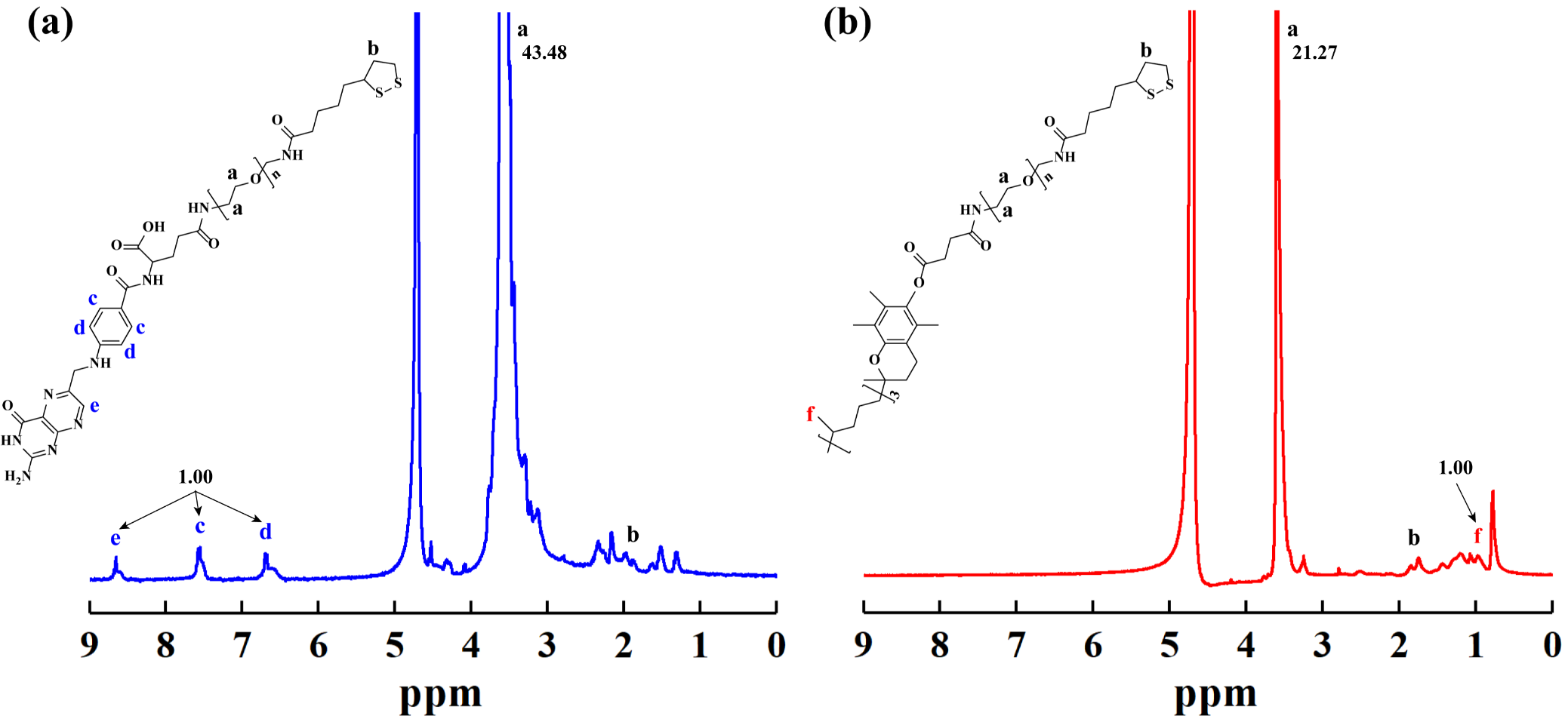


**Fig. S1** ^1^H NMR spectra of LA-PEG-FA and LA-PEG-TOS in D_2_O.





**Fig. S2** Hydrodynamic size of MoS_2_ nanoflakes and MPTF in water (n = 3). The error bar represents the standard deviation of data form 3 runs.





**Fig. S3** Surface potential of MoS_2_ nanoflakes and MPTF in water (n = 3). The error bar represents the standard deviation of data form 3 runs.

**Fig. S4** Temperature change (Δ*T*) of the aqueous solution of MPTF (1.0 mg/mL) under 808 nm laser irradiation with different laser powers for 300 s.





**Fig. S5** IC_50_ of α-TOS, PEG-TOS and MPTF for SKoV3 cells at 24 h or 48 h (n = 3).


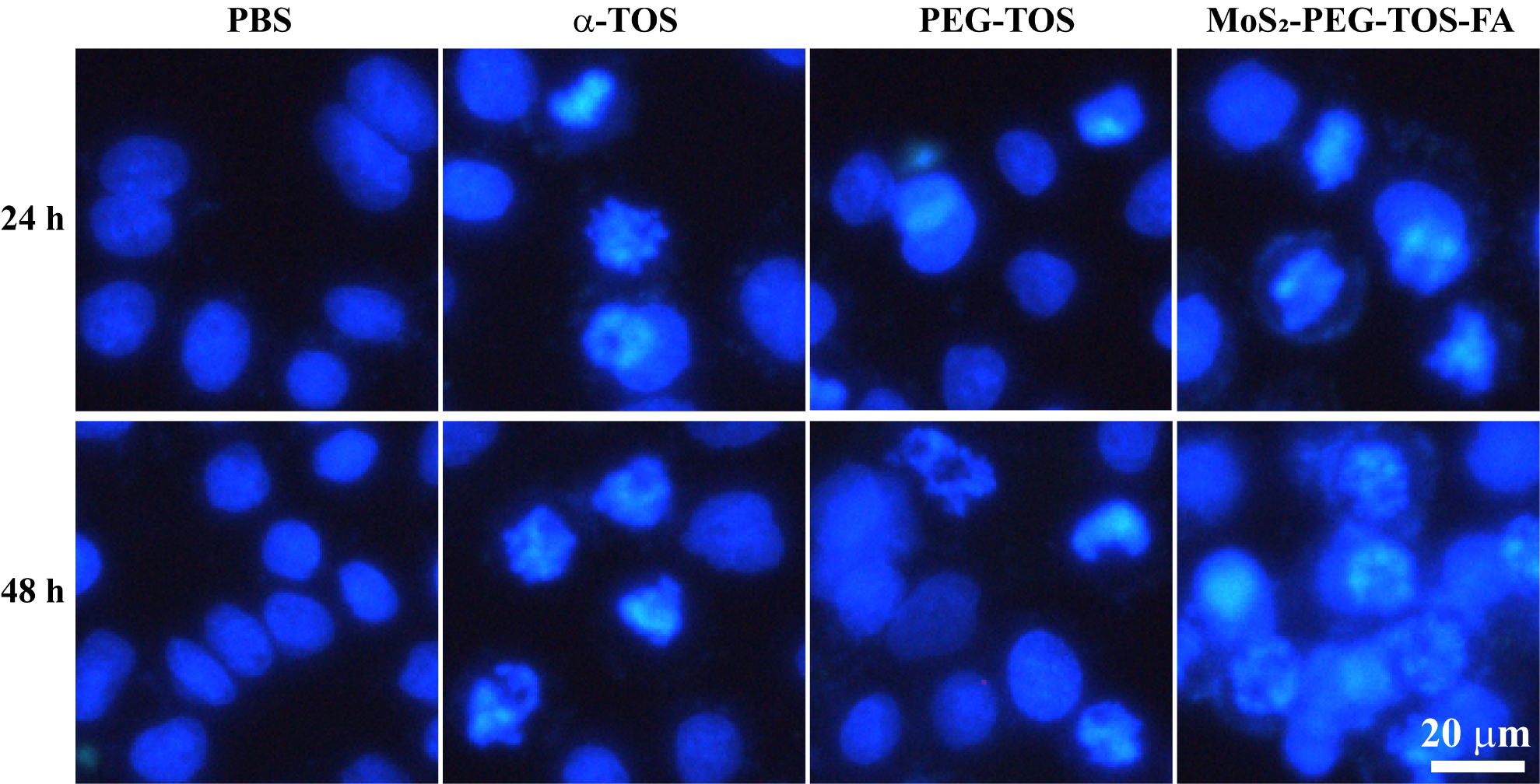


**Fig. S6** Fluorescence microscopic images of partially magnified SKoV3 cells (nuclei stained by Hoechst 33342) treated with PBS, α-TOS, PEG-TOS, MPTF for 24 h and 48 h.

**Fig. S7** CCK-8 assay of SKoV3 cell viability after exposed to various concentrations of MPF and MPTF with or without NIR irradiation (808 nm, 0.3 W/cm^2^) for 10 min.


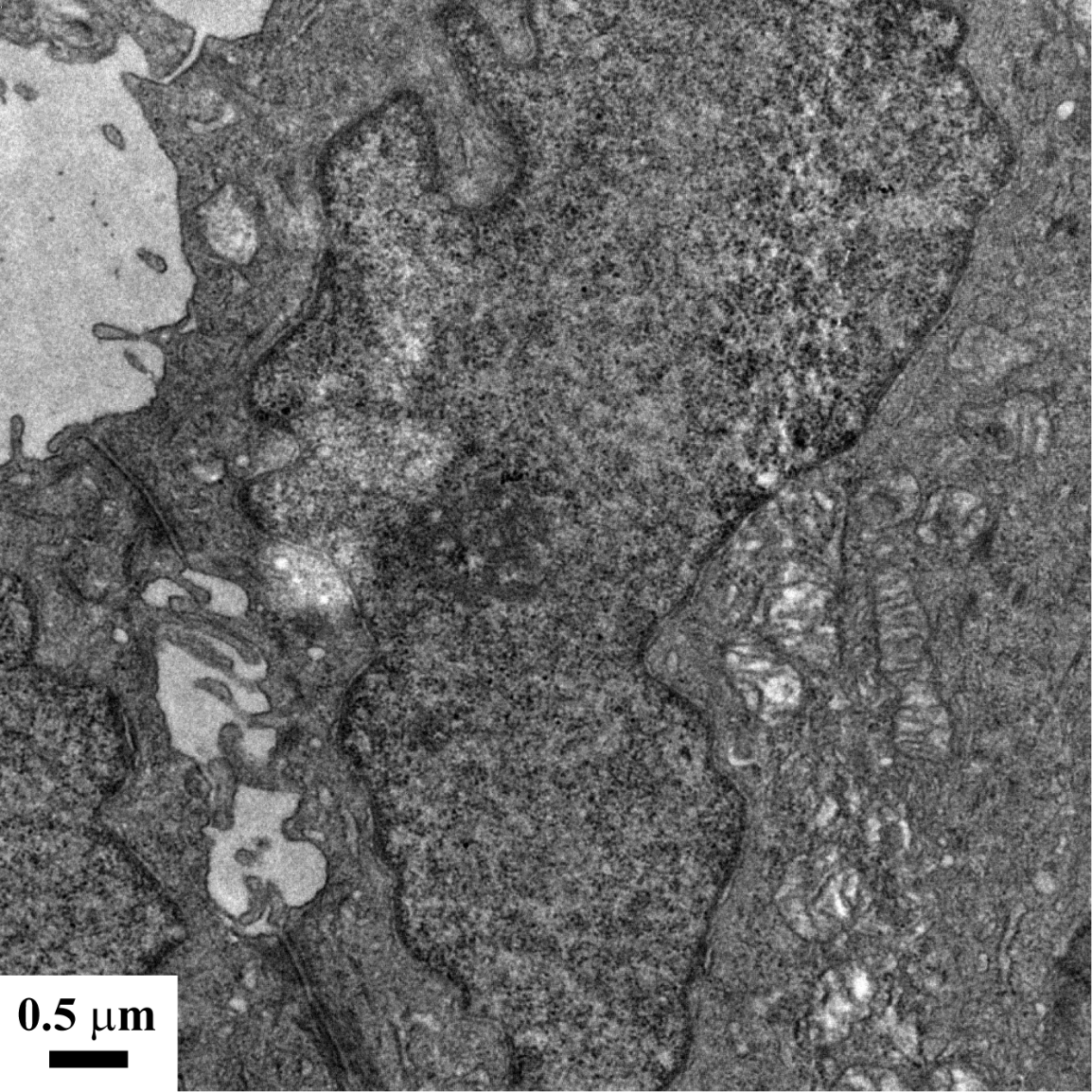


**Fig. S8** Bio-TEM image of tumor tissue obtained at 1 h after i.v. injection of saline.


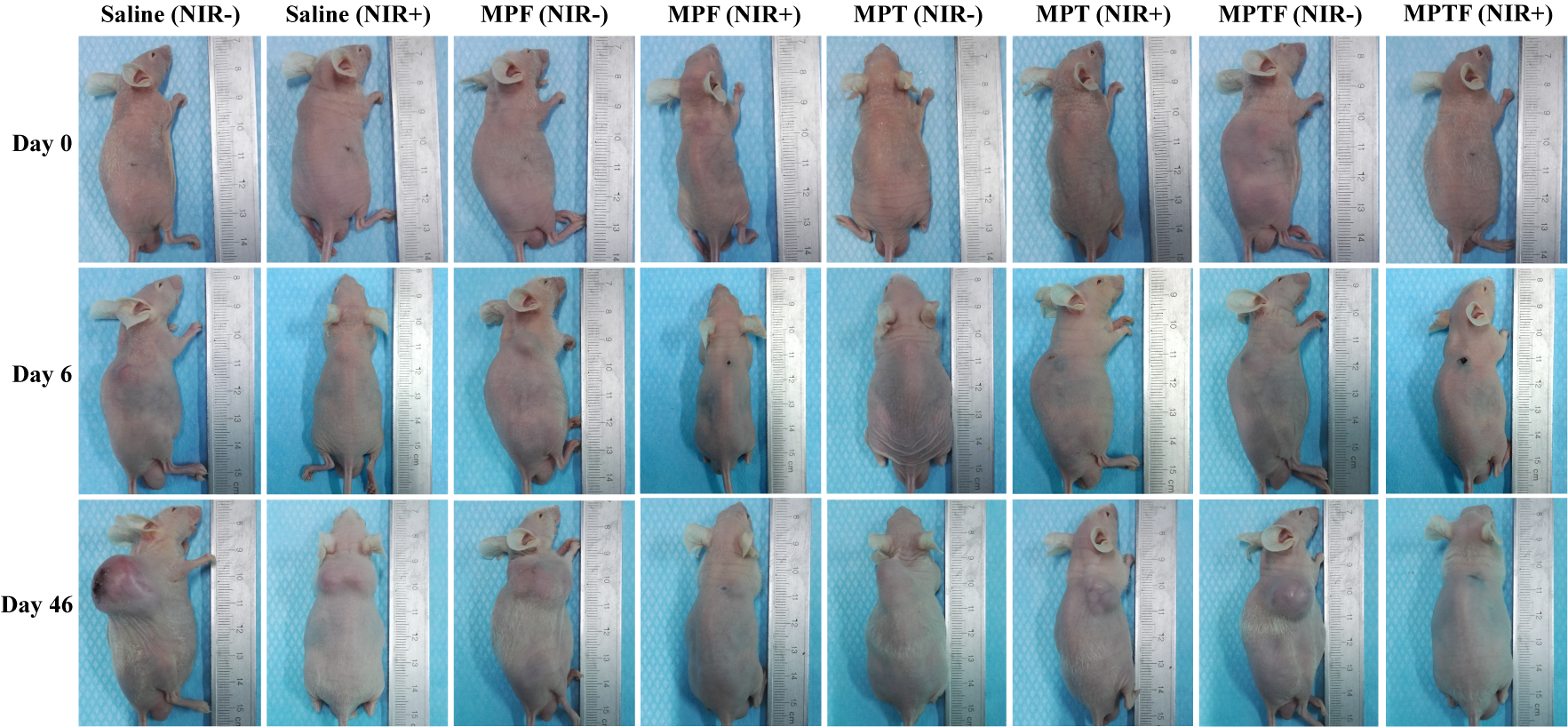


**Fig. S9** Digital photographs of the mice before and at day 6 and day 46 after different treatments.





**Fig. S10** Quantitative analysis of the apoptosis rate of tumor cells in different treatment groups.

**Fig. S11** Body weight change of the mice as a function of time in different treatment groups.


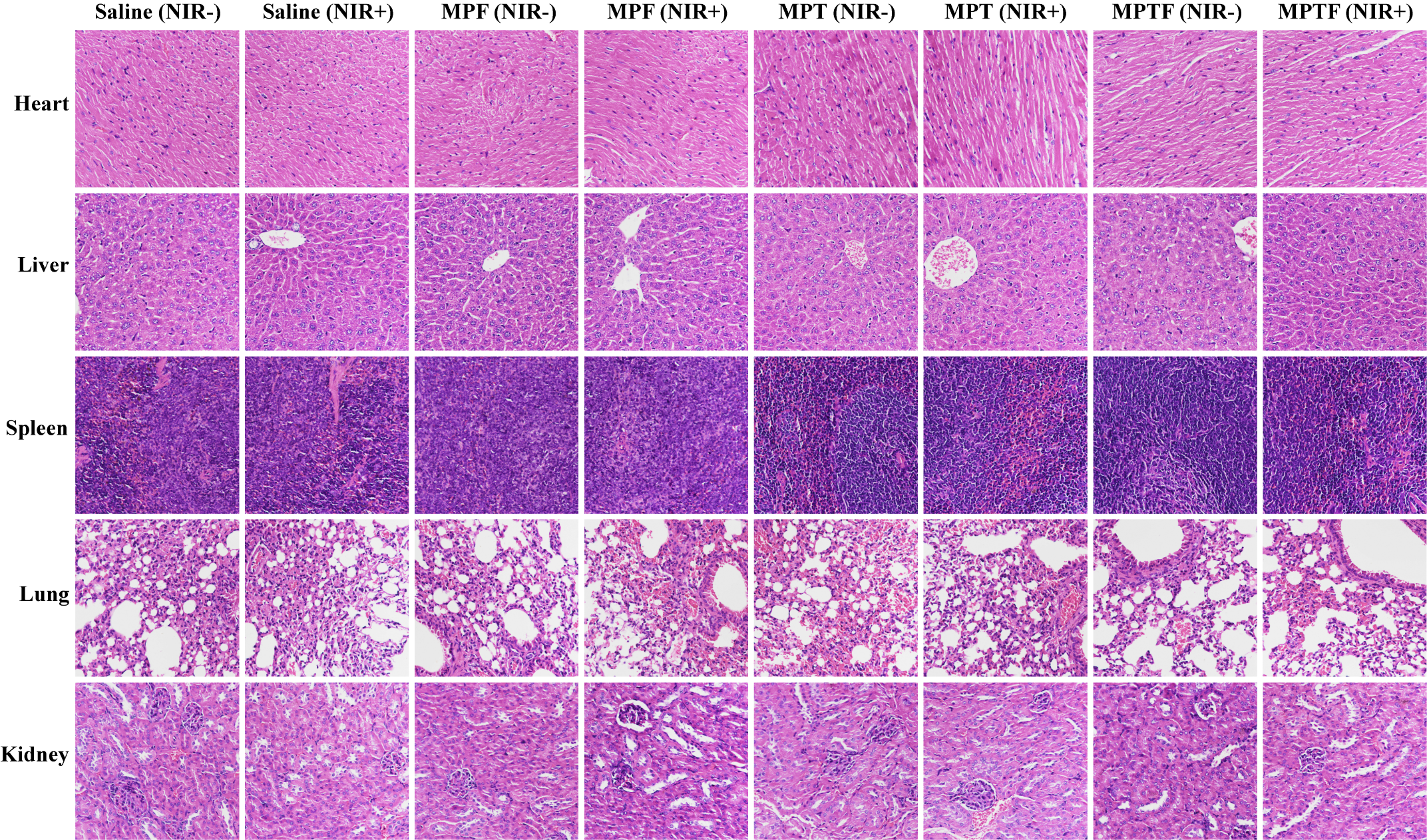


**Fig. S12** H&E staining of heart, liver, spleen, lung and kidney of mice in all groups at 46 days (×200).





**Fig. S13** Mo element amount in different organs of mice at different times postinjection of MPTF.





**Fig. S14** Mo levels in urine and feces collected at different times postinjection of MPTF.

**Table S1.** Comparison of photothermal therapy performance for reported agents under 808 nm laser irradiation (MPE: 0.33 W/cm^2^ for skin) *in vivo*.

| **Photothermal agents** | **PCE** | **Power** | **Administration ways** | **Reference** |
| --- | --- | --- | --- | --- |
| Ru@AuNPs | 18.3%-33.3% | 0.8 W/cm^2^ | i.t. | [3] |
| Gold nanoshells, nanorods or nanovesicles | 13%, 22% or 37% | 1 W/cm^2^ | i.t. | [4] |
| AuNSs-PEI or Au-PEI@pD NSs | 36.1% or 49.9% | 1.3 W/cm^2^ | i.t. | [5] |
| Fa-mPEG@CP5-CuS@HMSN-Py NPs | 19% | 1 W/cm^2^ | i.v. | [6] |
| SWNT-PEG | N.A. | 1 W/cm^2^ | i.t. | [7] |
| ICG-loaded MWCNTs | N.A. | 1 W/cm^2^ | i.v. | [8] |
| G5-MoS_2_/Bcl-2 siRNA polyplexes | N.A. | 1.2 W/cm^2^ | i.t. | [9] |
| PVP-Bi nanodots | 30% | 1.3 W/cm^2^ | i.v. | [10] |
| mPt@mSiO_2_-GdDTPA | 27% | 1.5 W/cm^2^ | i.t. | [11] |
| rGO-PEG/DOX | N.A. | 2 W/cm^2^ | i.v. | [12] |
| Cu_2-x_S:Pt/PVP NPs | 27%-37% | 2 W/cm^2^ | i.v. | [13] |
| DLQ/DZ | 21.9% | 2 W/cm^2^ | i.v. | [14] |
| IONP-TAT-Tf | 32%-43% | 3 W/cm^2^ | i.v. | [15] |
| NaLuF_4_: Gd/Yb/Er NRs@PDA | 40.18% | 3 W/cm^2^ | i.t. | [16] |
| PPy NPs | 44.7% | 1 W/cm^2^ | i.v. | [17] |
| γ-PGA/Cys@PANI NGs | N.A. | 1.5 W/cm^2^ | i.t. | [18] |
| Dpa-melanin CNSs | 40% | 2 W/cm^2^ | i.t. | [19] |
| Poly I:C-ICG@TRLs liposomes | N.A. | 1 W/cm^2^ | i.t. | [20] |
| WO@ICG | N.A. | 2 W/cm^2^ | i.t. | [21] |
| MoS_2_-PEG-TOS-FA | 65.3% | 0.3 W/cm^2^ | i.v. | This work |

**Table S2.** Equivalent relationship between IC_50_ concentration of α-TOS and sample concentration.

| **Sample** | **IC_50_ concentration (a-TOS)** | **Sample concentration** |
| --- | --- | --- |
| α-TOS 24 h | 42.3 μM | 22.5 μg/mL |
| α-TOS 48 h | 32.7 μM | 17.4 μg/mL |
| PEG-TOS 24 h | 35.4 μM | 89.5 μg/mL |
| PEG-TOS 48 h | 22.5 μM | 56.9 μg/mL |
| MPTF 24h | 17.5 μM | 416.5 μg/mL |
| MPTF 48h | 9.8 μM | 233.3 μg/mL |

**References**

1. Hu Y, Wang RZ, Wang SG, Ding L, Li JC, Luo Y*, et al.* Multifunctional Fe3O4@Au core/shell nanostars: A unique platform for multimode imaging and photothermal therapy of tumors. *Sci Rep* 2016; **6**: 28325.

2. Li X, Xing LX, Hu Y, Xiong ZJ, Wang RZ, Xu XY*, et al.* An RGD-modified hollow silica@Au core/shell nanoplatform for tumor combination therapy. *Acta Biomater* 2017; **62**: 273-83.

3. Zhang PY, Wang JQ, Huang HY, Yu BL, Qiu KQ, Huang JJ*, et al.* Unexpected high photothemal conversion efficiency of gold nanospheres upon grafting with two-photon luminescent ruthenium(II) complexes: A way towards cancer therapy? *Biomaterials* 2015; **63**: 102-14.

4. Huang P, Lin J, Li WW, Rong PF, Wang Z, Wang SJ*, et al.* Biodegradable gold nanovesicles with an ultrastrong plasmonic coupling effect for photoacoustic imaging and photothermal therapy. *Angew Chem, Int Ed* 2013; **52**: 13958-64.

5. Li D, Zhang YX, Wen SH, Song Y, Tang YQ, Zhu XY*, et al.* Construction of polydopamine-coated gold nanostars for CT imaging and enhanced photothermal therapy of tumors: an innovative theranostic strategy. *J Mater Chem B* 2016; **4**: 4216-26.

6. Yang J, Dai DH, Lou XY, Ma LJ, Wang BL, Yang YW. Supramolecular nanomaterials based on hollow mesoporous drug carriers and macrocycle-capped CuS nanogates for synergistic chemo-photothermal therapy. *Theranostics* 2020; **10**: 615-29.

7. Zhou FF, Wu SN, Wu BY, Chen WR, Xing D. Mitochondria-targeting single-walled carbon nanotubes for cancer photothermal therapy. *Small* 2011; **7**: 2727-35.

8. Hu Y, Wang RZ, Zhou YW, Yu N, Chen ZG, Gao DM*, et al.* Targeted dual-mode imaging and phototherapy of tumors using ICG-loaded multifunctional MWCNTs as a versatile platform. *J Mater Chem B* 2018; **6**: 6122-32.

9. Kong LD, Xing LX, Zhou BQ, Du LF, Shi XY. Dendrimer-modified MoS2 nanoflakes as a platform for combinational gene silencing and photothermal therapy of tumors. *ACS Appl Mater Interfaces* 2017; **9**: 15995-6005.

10. Lei PP, An R, Zhang P, Yao S, Song SY, Dong LL*, et al.* Ultrafast synthesis of ultrasmall poly(vinylpyrrolidone)-protected bismuth nanodots as a multifunctional theranostic agent for in vivo dual-modal CT/photothermal-imaging-guided photothermal therapy. *Adv Funct Mater* 2017; **27**: 1702018.

11. Zhao L, Ge XQ, Yan GH, Wang X, Hu PF, Shi LY*, et al.* Double-mesoporous core-shell nanosystems based on platinum nanoparticles functionalized with lanthanide complexes for in vivo magnetic resonance imaging and photothermal therapy. *Nanoscale* 2017; **9**: 16012-23.

12. Liu JJ, Liu K, Feng LZ, Liu Z, Xu LG. Comparison of nanomedicine-based chemotherapy, photodynamic therapy and photothermal therapy using reduced graphene oxide for the model system. *Biomater Sci* 2017; **5**: 331-40.

13. Dong LL, Zhang P, Xu X, Lei PP, Du KM, Zhang ML*, et al.* Simple construction of Cu2-xS: Pt nanoparticles as nanotheranostic agent for imaging-guided chemo-photothermal synergistic therapy of cancer. *Nanoscale* 2018; **10**: 10945-51.

14. Qiao JN, Tian FC, Deng YD, Shang YK, Chen SJ, Chang EH*, et al.* Bio-orthogonal click-targeting nanocomposites for chemo-photothermal synergistic therapy in breast cancer. *Theranostics* 2020; **10**: 5305-21.

15. Peng HB, Tang J, Zheng R, Guo GN, Dong AG, Wang YJ*, et al.* Nuclear-targeted multifunctional magnetic nanoparticles for photothermal therapy. *Adv Healthcare Mater* 2017; **6**: 1601289.

16. Li XL, Jiang MY, Zeng SJ, Liu HR. Polydopamine coated multifunctional lanthanide theranostic agent for vascular malformation and tumor vessel imaging beyond 1500 nm and imaging-guided photothermal therapy. *Theranostics* 2019; **9**: 3866-78.

17. Chen M, Fang XL, Tang SH, Zheng NF. Polypyrrole nanoparticles for high-performance in vivo near-infrared photothermal cancer therapy. *Chem Commun* 2012; **48**: 8934-6.

18. Zhou YW, Hu Y, Sun WJ, Zhou BQ, Zhu JZ, Peng C*, et al.* Polyaniline-loaded gamma-polyglutamic acid nanogels as a platform for photoacoustic imaging-guided tumor photothermal therapy. *Nanoscale* 2017; **9**: 12746-54.

19. Liu YL, Ai KL, Liu JH, Deng M, He YY, Lu LH. Dopamine-melanin colloidal nanospheres: An efficient near-infrared photothermal therapeutic agent for in vivo cancer therapy. *Adv Mater* 2013; **25**: 1353-9.

20. Xu L, Zhang W, Park HB, Kwak M, Oh J, Lee PCW*, et al.* Indocyanine green and poly I:C containing thermo-responsive liposomes used in immune-photothermal therapy prevent cancer growth and metastasis. *J Immunother Cancer* 2019; **7**: 220.

21. Deng KR, Hou ZY, Deng XR, Yang PP, Li CX, Lin J. Enhanced antitumor efficacy by 808 nm laser-induced synergistic photothermal and photodynamic therapy based on a indocyanine-green-attached W18O49 nanostructure. *Adv Funct Mater* 2015; **25**: 7280-90.
